# The orchestration of cell-cycle reentry and ribosome biogenesis network is critical for cardiac repair

**DOI:** 10.7150/thno.96460

**Published:** 2024-06-24

**Authors:** Yanli Wang, Junchu Tu, Weiliang Wu, Yan Xu, Yujie Li, Xiangbin Pan, Bin Liu, Tonggan Lu, Qingfang Han, Huiling Zhang, Lijuan Jiao, Yu Zhang, Xi-Yong Yu, Zhenya Shen, Yangxin Li

**Affiliations:** 1 Department of Cardiovascular Surgery of the First Affiliated Hospital & Institute for Cardiovascular Science, Collaborative Innovation Center of Hematology, State Key Laboratory of Radiation Medicine and Protection, Suzhou Medical College, Soochow University, Suzhou, Jiangsu 215123, P. R. China.; 2 Department of Geriatrics, The Second Xiangya Hospital, Central South University, Changsha, Hunan 410011, P. R. China.; 3 Department of Structural Heart Disease, National Center for Cardiovascular Disease, China & Fuwai Hospital, Chinese Academy of Medical Sciences & Peking Union Medical College, Beijing, Key Laboratory of Cardiovascular Apparatus Innovation, Beijing 100037, P. R. China.; 4 Department of Cardiology, the Second Hospital of Jilin University, Changchun, Jilin 130041, P. R. China.; 5 Key Laboratory of Molecular Target & Clinical Pharmacology and the NMPA State Key Laboratory of Respiratory Disease, Guangzhou Medical University, Guangzhou, Guangdong 511436, P. R. China.

**Keywords:** Ribosome biogenesis, circASXL1, Nucleolin, Exosome, Cardiac repair

## Abstract

**Rationale:** Myocardial infarction (MI) is a severe global clinical condition with widespread prevalence. The adult mammalian heart's limited capacity to generate new cardiomyocytes (CMs) in response to injury remains a primary obstacle in developing effective therapies. Current approaches focus on inducing the proliferation of existing CMs through cell-cycle reentry. However, this method primarily elevates cyclin dependent kinase 6 (CDK6) and DNA content, lacking proper cytokinesis and resulting in the formation of dysfunctional binucleated CMs. Cytokinesis is dependent on ribosome biogenesis (Ribo-bio), a crucial process modulated by nucleolin (Ncl).

Our objective was to identify a novel approach that promotes both DNA synthesis and cytokinesis.

**Methods:** Various techniques, including RNA/protein-sequencing analysis, Ribo-Halo, Ribo-disome, flow cytometry, and cardiac-specific tumor-suppressor retinoblastoma-1 (Rb1) knockout mice, were employed to assess the series signaling of proliferation/cell-cycle reentry and Ribo-bio/cytokinesis. Echocardiography, confocal imaging, and histology were utilized to evaluate cardiac function.

**Results:** Analysis revealed significantly elevated levels of Rb1, bur decreased levels of circASXL1 in the hearts of MI mice compared to control mice. Deletion of Rb1 induces solely cell-cycle reentry, while augmenting the Ribo-bio modulator Ncl leads to cytokinesis. Mechanically, bioinformatics and the loss/gain studies uncovered that circASXL1/CDK6/Rb1 regulates cell-cycle reentry. Moreover, Ribo-Halo, Ribo-disome and circRNA pull-down assays demonstrated that circASXL1 promotes cytokinesis through Ncl/Ribo-bio. Importantly, exosomes derived from umbilical cord mesenchymal stem cells (UMSC-Exo) had the ability to enhance cardiac function by facilitating the coordinated signaling of cell-cycle reentry and Ribo-bio/cytokinesis. These effects were attenuated by silencing circASXL1 in UMSC-Exo.

**Conclusion:** The series signaling of circASXL1/CDK6/Rb1/cell-cycle reentry and circASXL1/Ncl/Ribo-bio/cytokinesis plays a crucial role in cardiac repair. UMSC-Exo effectively repairs infarcted myocardium by stimulating CM cell-cycle reentry and cytokinesis in a circASXL1-dependent manner. This study provides innovative therapeutic strategies targeting the circASXL1 signaling network for MI and offering potential avenues for enhanced cardiac repair.

## Introduction

Myocardial infarction (MI) is a critical clinical condition affecting numerous patients globally 1. The adult mammalian heart faces a significant hurdle in generating new cardiomyocytes (CMs) following injury, posing a primary challenge for cardiac repair 2. One of the existing therapeutic approaches involves inducing the proliferation of existing CMs through cell-cycle reentry 3. However, this strategy tends to elevate DNA content without proper cytokinesis, leading to the formation of dysfunctional binucleated CMs 4. In this study, our objective was to uncover a novel approach that promoting both cell-cycle reentry and cytokinesis while elucidating the molecular mechanisms.

Cytokinesis is regulated by ribosome biogenesis (Ribo-bio) 5. Sustaining efficient Ribo-bio necessitates elevated levels of pre-47S rRNA, a crucial ribosome component transcribed by RNA polymerase I (Pol I) 6. Nucleolin (Ncl), a regulator of Ribo-bio, involved in various physiological and pathological conditions 7, 8. However, the specific roles of pre-47S rRNA and Ncl in cardiac repair remain unclear.

Due to the heightened regenerative potential observed in CMs from young mice, we conducted RNA/protein sequencing to compare hearts from young and old mice. The aim was to uncover novel pathways in cardiac repair that regulate both CMs cell-cycle reentry and cytokinesis. The data revealed elevated levels of retinoblastoma-1 (Rb1) and cyclin-dependent kinase inhibitor 2a (CDKN2a), accompanied by reduced levels of pre-47S rRNA, Ncl, and Pol I in the hearts of aged mice. Rb1 protein is a well-known tumor-suppressor, with its inactivation known to promote tumor cell proliferation 9, 10. In this context, we explored the roles of Rb1, pre-47S rRNA, and Ribo-bio modulator Ncl in cardiac repair.

Circular RNAs (circRNAs) are non-coding RNAs (ncRNAs) lacking a 5' cap and 3' poly (A) tail, rendering them more stable than other ncRNAs 11, 12. These circRNAs, known for their tissue-specificity, play crucial roles in the cardiovascular system 13-15. Our newly acquired circRNA data highlight circASXL1 as one of the most abundant circRNAs in the hearts of young mice, with its expression markedly reduced in the aging and MI heart. Through bioinformatics analysis, we identified a potential downstream signaling pathway of circASXL1 involving miR-1/cyclin-dependent kinase 6 (CDK6)/cell-cycle reentry. Previous studies have established that CDK6 enhance cell proliferation by inactivating Rb1 16. Furthermore, bioinformatics analysis unveiled Ncl as a potential direct target of circASXL1. Consequently, our investigation aims to elucidate whether circASXL1 can modulate both miR-1/CDK6/cell-cycle reentry and Ncl/Ribo-bio/cytokinesis in cardiac repair following MI.

Exosomes, microvesicles harboring non-coding RNA (ncRNAs) like circRNA, are released by various cell types 17. Previous studies, including our own, have demonstrated that exosomes-derived from human umbilical cord mesenchymal stem cells (UMSC-Exo) can transport miRNA and circRNA to facilitate the repair of ischemic tissues 18-21. Yet, it remains unclear whether UMSC-Exo can augment cardiac repair by releasing circASXL1 to modulate processes like circASXL1/CDK6/Rb1/cell-cycle reentry and circASXL1/Ncl/Ribo-bio.

In this investigation, we employed discovery-driven methodologies, encompassing circRNA/mRNA/protein sequencing, Ribo-Halo, Ribo-disome, circRNA pull-down assay, and a cardiac-specific Rb1-knockout mouse model. Through these approaches, we elucidated the crucial involvement of the circASXL1/Rb1/cell-cycle reentry axis and the circASXL1/Ncl/Ribo-bio signaling pathway in MI. Additionally, we sought to determine whether circASXL1 mediates the beneficial effects of UMSC-Exo in facilitating CM cell-cycle reentry and cytokinesis.

## Results

### Identification of the Rb1/cell-cycle reentry signaling network through mRNA/protein-sequencing

To unravel key molecules and potential signaling network triggering cardiac regeneration, we conducted a comparative analysis of mRNA transcripts and protein expression profiles in young and aging hearts using RNA sequencing (RNA-seq) and proteomic sequencing. The decision to compare young versus aging hearts, rather than normal and infarcted hearts, was driven by the superior regenerative potential of CMs from young mice.

**Figure 1:**
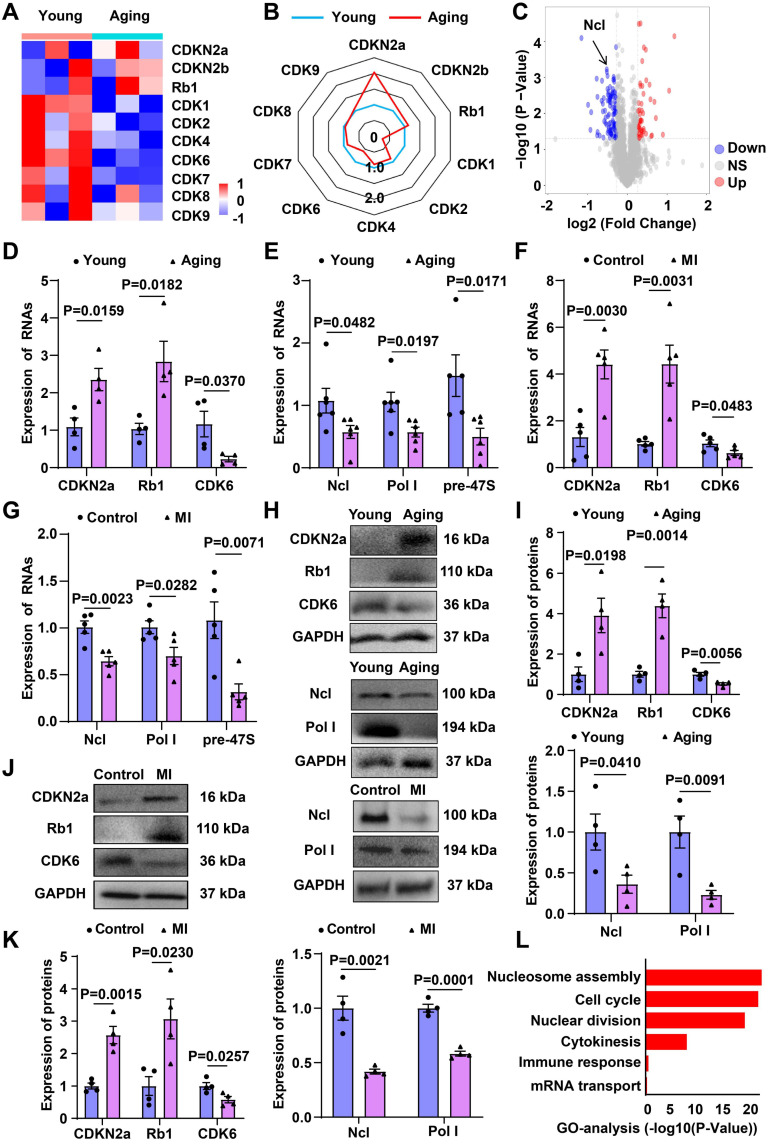
** mRNA and proteome sequencing of aging and young heart.** (**A**) Heatmap of mRNA sequencing data from the hearts of aging and young mice (blue, downregulated; red, upregulated). (**B**) Radar chart of mRNA sequencing data (higher number indicates upregulation). (**C**) Volcano map for proteome sequencing of aging and young mice (blue, downregulated; red, upregulated). (**D**) RT-qPCR analysis of mRNA levels of CDKN2a, Rb1 and CDK6 comparing aging and young heart (n=4). (**E**) RT-qPCR analysis of mRNA levels of Ncl, Pol Ι and pre-47S rRNA comparing aging and young heart (n=6). (**F**) RT-qPCR analysis of mRNA levels of CDKN2a, Rb1 and CDK6 comparing control and MI (n=5). (**G**) RT-qPCR analysis of mRNA levels of Ncl, Pol Ι and pre-47S rRNA comparing control and MI (n=5). (**H, I**) Western blot analysis of the expression of Rb1, CDKN2a, Ncl, Pol Ι and CDK6 in young and aging mice (n=4). (**J, K**) Western blot analysis of the expression of Rb1, CDKN2a, Ncl, Pol Ι and CDK6 in control and MI mice (n=4). (**L**) GO term enrichment of differentially regulated genes in the aging heart compared with the control group. Data are presented as the mean±SEM.

RNA-seq revealed increased mRNA levels of Rb1 and CDKN2a in aging hearts, while CDK6 mRNA levels were decreased (Figure 1A and 1B). Proteomic sequencing demonstrated a significant decrease in the protein levels of Ncl in aging hearts (Figure 1C). RT-qPCR confirmed the upregulation of Rb1, CDKN2a, and the downregulation of Ncl, pre-47S rRNA, RNA Pol І, CDK6 in both aging and infarcted hearts (Figure 1D-1G). Western blot analysis further validated the increased expression of Rb1 and CDKN2a, as well as the decreased expression of Ncl, RNA Pol І, CDK6 in aging and infarcted hearts (Figure 1H-1K).

The differentially expressed mRNAs were subjected to gene ontology (GO) categories and pathway analysis, revealing significant alterations in biological processes, including cell cycle, immune response, nuclear division, cytokinesis, nucleosome assembly, and mRNA transport in aging hearts (Figure 1L). The involvement of cell-cycle reentry and cytokinesis-related signaling network in the aging process provides valuable insights into the potential mechanisms underlying cardiac regeneration.

To validate RNA-seq data, a portion of the same samples used for RNA sequencing underwent protein sequencing (Figure S1A). Heat map analysis demonstrated a correlation between genes and proteins in young and aging hearts (Figure S1B). Notably, spearman's correlation coefficients showed significant changes in the expression of Rb1, Ncl, and CDK6 between aging and young hearts at both mRNA and protein levels (Figure S1C-1E). ceRNA-GO analysis further confirmed significant alterations in cell proliferation and cytokinesis in aging hearts (Figure S1F). These findings prompted further exploration of the role of cell-cycle reentry and cytokinesis in cardiac regeneration.

### Decreased expression of circASXL1 in MI

In searching circRNAs potentially implicated in cardiac regeneration, we conducted a comparative analysis of circRNA expression in CMs using RT-qPCR. Notably, circASXL1 exhibited significantly higher expression than other circRNAs (Figure S2A) and demonstrated a reduction in expression in the aging heart (Figure S2B). Importantly, we observed a decline in circASXL1 expression in the myocardium following MI (Figure S2C). Derived from Exon 2, 3, and 4 of the ASXL1 gene (Figure S2D), circASXL1's circRNA nature was confirmed through divergent PCR and subsequent Sanger sequencing analysis (Figure S2E and 2F). In addition, we also found that circASXL1 is expressed in cardiac fibroblasts (CFs), vascular smooth muscle cells (VSMCs) (Figure S3A-3D), and their exosomes (Figure S3E-3J). More importantly, we analyzed the expression of circASXL1 in CFs on day 1 and day 3 after MI, and the results indicated no significant changes (Figure S3K and 3L). These findings suggest that circASXL1 may play a pivotal role in MI development.

### Promotion of cardiac repair by exosomal circASXL1

Previous research has demonstrated that the positive impact of exosomes derived from stem cells on cardiac repair is attributed to ncRNAs 22, 23. In our study, we isolated exosomes from UMSCs and found enrichment of circASXL1 in both UMSCs and exosomes (Figure S4A-4E).

**Figure 2:**
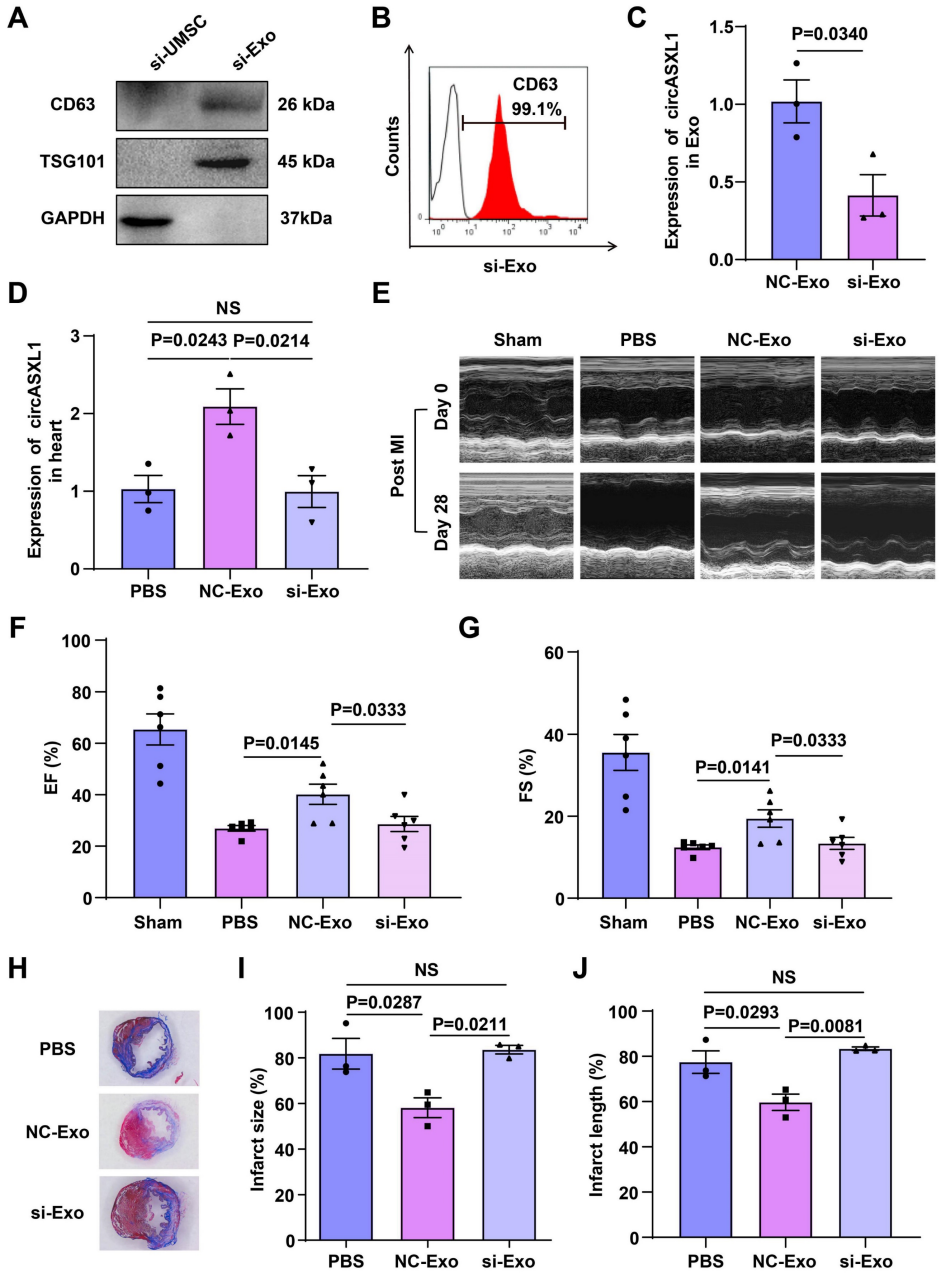
** UMSC-Exo-derived circASXL1 promotes cardiac repair.** (**A, B**) The expression of exosomal marker CD63 and TSG101 in UMSCs transfected with si-circASXL1(si-UMSC), and exosomes isolated from these cells (si-Exo) was determined by Western blots and flow cytometry. (**C**) RT-qPCR analysis of circASXL1 in NC-Exo and si-Exo (n=3). (**D**) RT-qPCR analysis of the expression of circASXL1 in ischemic myocardial tissue treated with PBS, NC-Exo, and si-Exo (n=3). (**E-G**) Cardiac function in different treatment groups was assessed by echocardiography (n=6). (**H-J**) Cardiac fibrosis was evaluated by Masson's trichrome-staining of transverse sections of infarcted myocardium (n=3). Data are presented as the mean±SEM.

To investigate whether circASXL1 is a key component of circRNAs responsible for these beneficial effects, we silenced circASXL1 in UMSCs, generating exosomes deficient in circASXL1 (si-Exo). Western blot and flow cytometry analyses revealed that circASXL1 silencing did not alter the expression of the exosome markers CD63 and TSG101 (Figure 2A and 2B) but significantly decreased circASXL1 expression in exosomes (Figure 2C). To evaluate the impact of circASXL1 knockdown *in vivo*, mice were treated with phosphate-buffered saline (PBS), control exosomes (NC-Exo), and si-Exo. NC-Exo treatment led to an increased expression of circASXL1 in the heart, a response attenuated by si-Exo (Figure 2D). Echocardiography conducted at day 28 post-MI revealed significantly higher left ventricular ejection fraction (EF) and fractional shortening (FS) in the NC-Exo group compared to the si-Exo group after MI (Figure 2E to 2G, Table S1). The assessment of infarction size using Masson's trichrome staining on thin myocardial sections demonstrated an increased percentage of fibrotic area in the entire cross-sectional area and the percentage of fibrosis length in the entire internal circumference in the si-Exo group compared to the NC-Exo group (Figure 2H-2J). We evaluated the heart weight-to-body weight ratio data following MI in the treated animals. Exosomes can attenuate cardiac hypertrophy and halt the heart failure in the long term (Figure S5A-5F). These findings suggest that circASXL1 plays a pivotal role in mediating the beneficial effects of UMSC-Exo on cardiac repair. Meanwhile, we analyzed the cell proliferation and biological functions of various cardiac cell types across the treatment groups (PBS, NC-Exo and si-Exo). Our data showed that exosomes promote VSMC proliferation but have no significant effect on CF proliferation (Figure S6A-6F). Furthermore, exosomes promote angiogenesis and reduce inflammation in infarcted myocardium (Figure S6G and 6H). We also examined the impact of these treatments on the structures and functions of other non-cardiac organs. Our data indicated that exosomes do not have a significant impact on other organs (Figure S7A-7L).

### Regulation of cell-cycle reentry by circASXL1 through the miR-1/CDK6 pathway

Micro-RNA-1 (miR-1) has been recognized for its significant role in the development of cardiovascular diseases 24. Utilizing CircBank analysis, we identified an interaction between miR-1 and circASXL1 (Figure S8A). This interaction was validated through a dual luciferase assay, demonstrating a robust binding of miR-1 to circASXL1 (Figure S8B). The expression of miR-1 was increased in the early post-MI phase (Figure S8C), contrasting with the expression pattern of circASXL1, as illustrated in Figure S2C. These findings suggest that circASXL1 serves as a molecular sponge for miR-1.

Consistent with previous reports indicating miR-1 targeting of CDK6 25, our bioinformatics analysis identified CDK6 as a target of miR-1, which was confirmed by a dual-luciferase reporter assay (Figure S8D and 8E). RT-qPCR analysis demonstrated that the expression of circASXL1 and CDK6 was downregulated by sh-circASXL1 (Figure S8F), while the expression of circASXL1 and CDK6 was upregulated by OE-circASXL1 (Figure S8G). Western blot results indicated a decrease in CDK6 expression in CMs treated with sh-circASXL1 (Figure S8H and 8I). These data collectively suggest that circASXL1 regulates cell-cycle reentry through the miR-1/CDK6 pathway.

### Promotion of CM cell-cycle reentry through Rb1 inhibition

**Figure 3:**
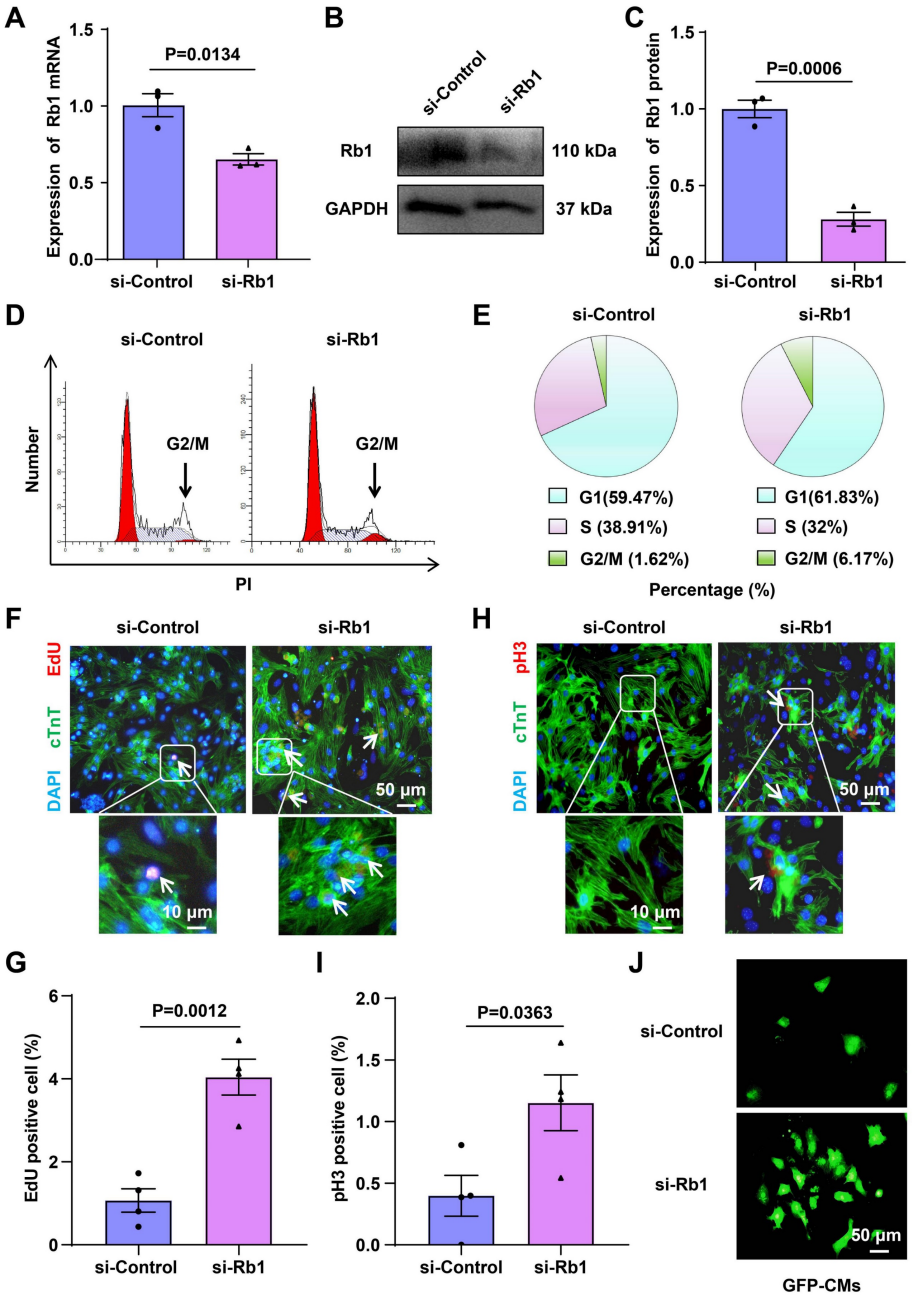
** Inhibition of Rb1 expression promotes CMs proliferation.** (**A-C**) RT-qPCR, and Western blot analysis of Rb1 expression in CMs transfected with siRNA targeting Rb1 (si-Rb1) or control (si-Control) (n=3). (**D, E**) Flow cytometry analysis of CMs proliferation (n=5). (**F, G**) The proliferation of CMs was detected by EdU incorporation. The cells were pre-treated with si-Control or si-Rb1. Blue: nuclear staining (DAPI); Red: EdU staining (scale bar: 50 μm) (n=4). (**H, I**) The proliferation of CMs was detected by pH3 immunofluorescence. Blue: nuclear staining (DAPI); Red: pH3 staining (scale bar: 50 μm) (n=4). (**J**) CMs from GFP mice were treated with si-Control or si-Rb1 (n=3). Data are presented as the mean±SEM.

To investigate the impact of Rb1 on cell-cycle reentry, primary CMs were isolated from 1-day-old C57BL mice, and the knockdown of Rb1 using siRNA-Rb1 (si-Rb1) in these primary CMs was confirmed through RT-qPCR and Western blot analysis (Figure 3A-3C). Flow cytometry-based cell cycle analysis demonstrated an increase in the percentage of cells in G2/M from 1.62% to 6.17% following treatment with si-Rb1 (Figure 3D and 3E). Enhanced DNA synthesis, as evidenced by 5-Ethynyl-2'-deoxyuridine (EdU) incorporation and an increased G2/M phase (pHistone 3 immunofluorescence), corroborated the occurrence of cell-cycle reentry in si-Rb1-treated cells (Figure 3F-3I). Moreover, si-Rb1 treatment led to an increased number of CMs isolated from 1-day-old GFP mice (Figure 3J), and it promoted the proliferation of H9C2 cells (Figure S9A-9E). These findings collectively suggest that inhibiting Rb1 expression promotes CMs cell-cycle reentry.

**Figure 4:**
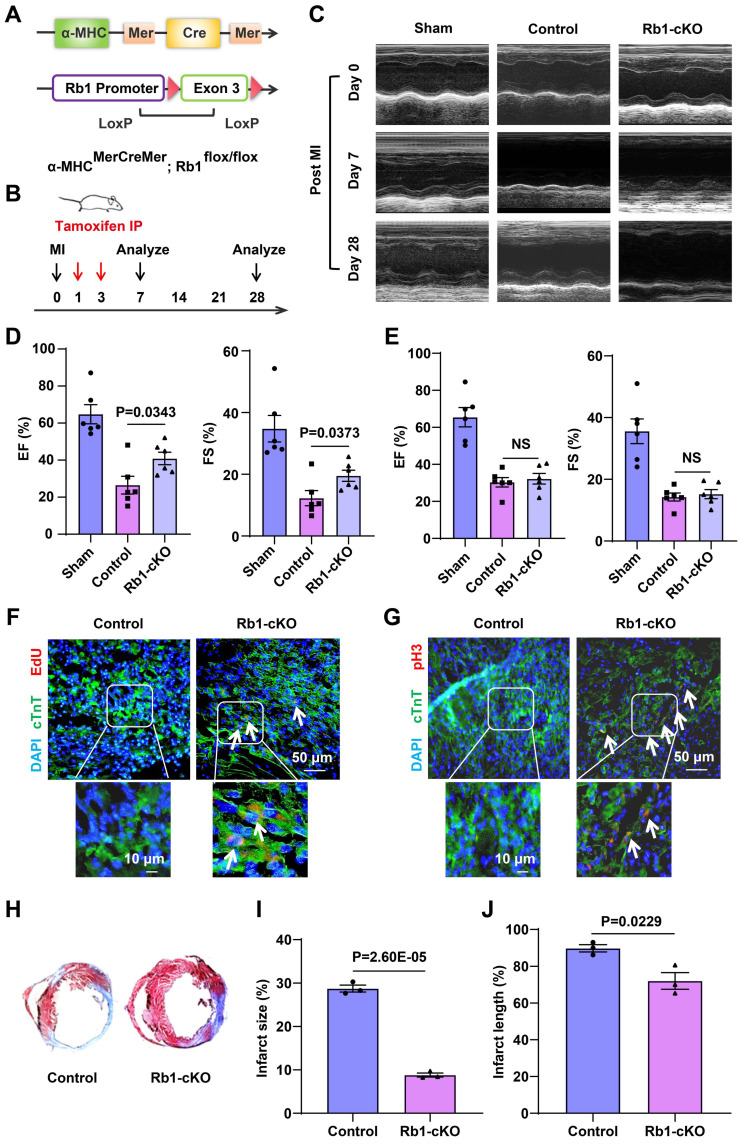
** Rb1 gene deletion has no long-term benefit for MI.** (**A**) Schematic illustration of the procedure to generate cardiac-specific knockout Rb1 mice. (**B**) Schematic diagram of MI model in Rb1 KO mice. (**C-E**) Cardiac function was assessed by echocardiography at 7 (**D**) and 28 (**E**) days post-injury (n=6). (**F**) EdU was injected into the intraperitoneal cavity of the mice. The proliferation of CMs was detected by EdU immunofluorescence on frozen sections of the myocardium (n=5). (**G**) The proliferation of CMs was detected by pH3 immunofluorescence on frozen sections of the myocardium (n=5). (**H-J**) Cardiac fibrosis was assessed by Masson's trichrome-staining of transverse sections of infarcted myocardium (n=3). Data are presented as the mean±SEM.

To further investigate the* in vivo* impact of Rb1 on CMs cell-cycle reentry*,* we generated cardiac-specific Rb1-cKO mice by crossing α-MHC^MerCreMer^ mice with Rb1^flox/Wt^ mice. Briefly, α-MHC^MerCreMer^ mice were mated with Rb1^flox/Wt^ mice to generate α-MHC^MerCreMer^ Rb1^flox/Wt^ heterozygous mice, which were then mated with each other to obtain cardiac-specific Rb1-cKO mice (Figure 4A and 4B). Genotyping was confirmed using specific primers (Figure S10A-10C). The Rb1-cKO mice exhibited comparable appearance and cardiac function to wild-type mice (Figure S10D-10F). Following tamoxifen induction, there was a significant reduction in the expression of Rb1 (Figure S10G). These data suggest that our Rb1-cKO mouse is a suitable model to evaluate the function of Rb1 in CMs cell-cycle reentry.

The Rb1-cKO mice demonstrated better cardiac functional recovery than wild-type mice on day 7 post-MI, as evidenced by improved ejection fraction (EF) and fractional shortening (FS) (Figure 4C and 4D, Table S2). However, no significant recovery was observed on day 28 (Figure 4E, Table S3). Increased EdU incorporation and pHistone 3 immunofluorescence confirmed CMs cell-cycle reentry in Rb1-cKO mice on day 7 (Figure 4F and 4G). Additionally, the Rb1-cKO mice exhibited lower percentages of fibrotic area in the entire cross-sectional area and fibrosis length in the entire internal circumference on day 7 (Figure 4H-4J). These data indicate that Rb1 deletion promotes CMs cell-cycle reentry and improves cardiac function, particularly at an early time point post-infarction. Additionally, we injected circASXL1 shRNA intramyocardialy to assess the impact of circASXL1 on cardiomyocyte proliferation in vivo. Our data show that the knockdown of circASXL1 reduced the proliferation of cardiomyocytes and cardiac function in Rb1-cKO mice compared with the control shRNA (Figure S11A-11E).

### circASXL1 enhances cytokinesis through ribo-bio regulation

While Rb1 knockdown alone did not fully replicate the beneficial effect of Exo-derived circASXL1, it suggested that circASXL1 might activate additional pathways in cardiac regeneration. Cytokinesis, a crucial process in cardiac regeneration, is regulated by Ribo-bio 26, 27, and is under control of Ncl 28.

**Figure 5:**
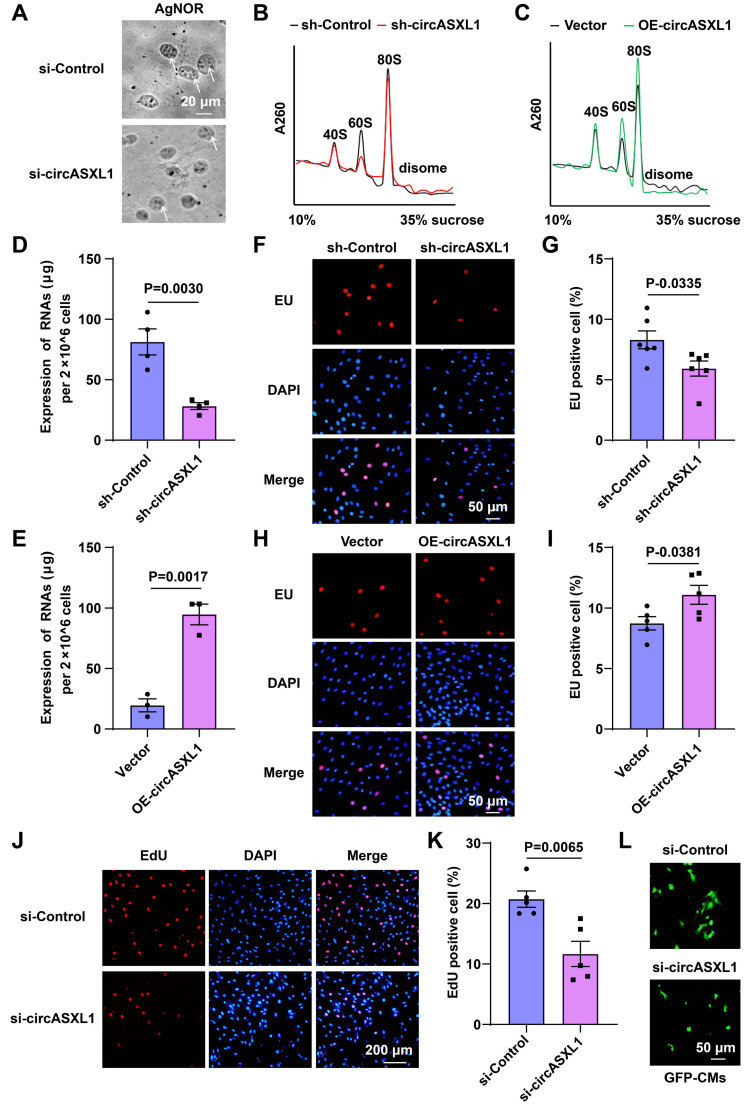
** circASXL1 promotes Ribo-bio and proliferation of H9C2 cell.** (**A**) The Ribo-bio of H9C2 (rat CMs) was detected by AgNOR staining (n=5). (**B, C**) Fractions from sucrose gradients (10%-35%) of lysates from H9C2 with circASXL1 overexpression or knockdown were collected (n=3). (**D, E**) The total RNA content of H9C2 was measured as an index of ribosomal content (since ribosomal RNA comprises over 85% of the total RNA pool) (n=4 or 3). (**F, G**) The rRNA synthesis of H9C2 was detected by EU incorporation. The cells were pre-treated with sh-Control or sh-circASXL1. Blue: nuclear staining (DAPI); Red: EU staining (scale bar: 50 μm) (n=6). (**H, I**) The rRNA synthesis of H9C2 was detected by EU incorporation. The cells were pre-treated with Vector or OE-circASXL1. Blue: nuclear staining (DAPI); Red: EU staining (scale bar: 50 μm) (n=5). (**J, K**) The proliferation of H9C2 was detected by EdU incorporation. The cells were pre-treated with si-Control or si-circASXL1. Blue: nuclear staining (DAPI); Red: EdU staining (scale bar: 200 μm) (n=5). (**L**) The primary CMs from GFP mice were treated with si-Control or si-circASXL1 (n=3). Data are presented as the mean±SEM.

To verify that circASXL1 promotes Ribo-bio and cell proliferation in CMs, we found that circASXL1 siRNA treatment reduced Ribo-bio in H9C2, as indicated by AgNOR staining (Figure 5A). Ribosome collisions were directly monitored by analyzing cellular lysates on a sucrose gradient, revealing that circASXL1 shRNA treatment decreased ribosome content, while circASXL1 overexpression had the opposite effect (Figure 5B and 5C). The expression of rRNA, assessed through RT-qPCR analysis and EU incorporation, was down-regulated by sh-circASXL1 and up-regulated by OE-circASXL1, confirming the influence of circASXL1 on ribosomal RNA content (Figure 5D-5I). The decrease in EdU incorporation following si-circASXL1 treatment corroborated the reduced proliferation of CMs (Figure 5J and 5K), and si-circASXL1 also reduced the number of CMs isolated from 1-day-old GFP mice (Figure 5L).

**Figure 6:**
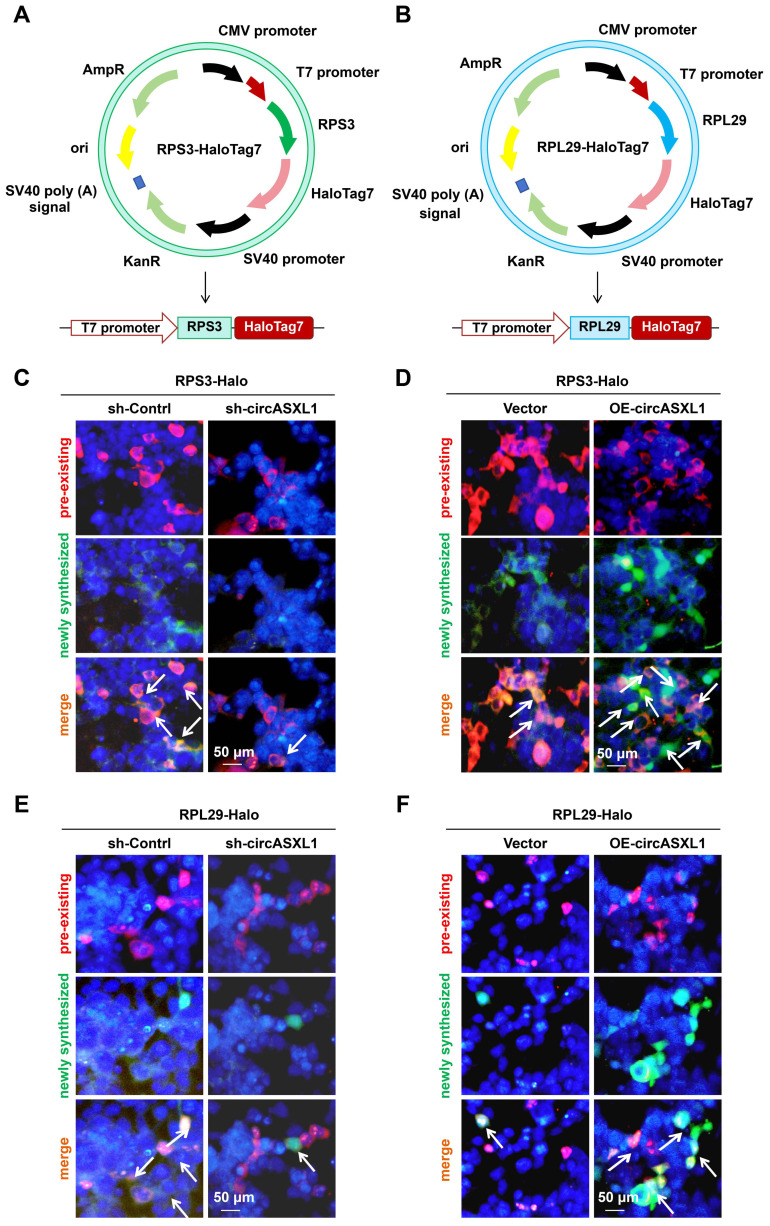
** Promotion of Ribo-bio by circASXL1 was identified through Ribo-Halo analysis.** (**A**) Schematic illustration of RPS3-HaloTag7 plasmid construction. (**B**) Schematic illustration of RPL29-HaloTag7 plasmid construction. (**C, D**) The Ribo-bio was detected by RRS3-Halo. The 293T cells were pre-treated with sh-Control, sh-circASXL1, or vector plasmid and circASXL1-overexpression plasmid. Blue: nuclear staining (DAPI); (scale bar: 50 μm) (n=5). (**E, F**) The Ribo-bio was detected by RRL29-Halo. The 293T cells were pre-treated with sh-Control, sh-circASXL1, or vector plasmid and circASXL1-overexpression plasmid. Blue: nuclear staining (DAPI); (scale bar: 50 μm) (n=5).

To assess new R-protein (ribosomal proteins) synthesis, Halo cassette (Halotag7) was fused to the C-termini of RPS3 and RPL29 genes (Figure 6A and 6B, Figure S12A). The Halo protein was tagged at the C terminus of RPS3 and RPL29 as the indicated R-proteins contain exposed C-termini and are situated at a considerable distance from the peptide exit tunnel, as evidenced by the structure of an 80S complex (Figure S12B).

As documented previously, Halo-fused proteins can undergo covalent labeling using distinct fluorescent ligands, facilitating the examination of new R-protein synthesis through imaging 29. In order to concurrently assess pre-existing R-proteins and the synthesis of new R-proteins, 293T Ribo-Halo cells were exposed to a red Halo ligand for 1 hour to label preexisting R-proteins. Subsequently, the cells were cultured in media containing a green Halo ligand for 24 hours to label newly translated R-proteins. The results indicated that circASXL1 promotes Ribo-bio by enhancing the synthesis of new R-proteins (Figure 6C to 6F, Figure S12C-12F). Interestingly, we found that silent Rb1 did not affect Ribo-bio (Figure S13A-13G). The results suggest that Rb1 knockdown alone cannot regulate Ribo-bio, and support our finding that Rb1 knockout mice improves cardiac function only at an early time point post-infarction.

**Figure 7:**
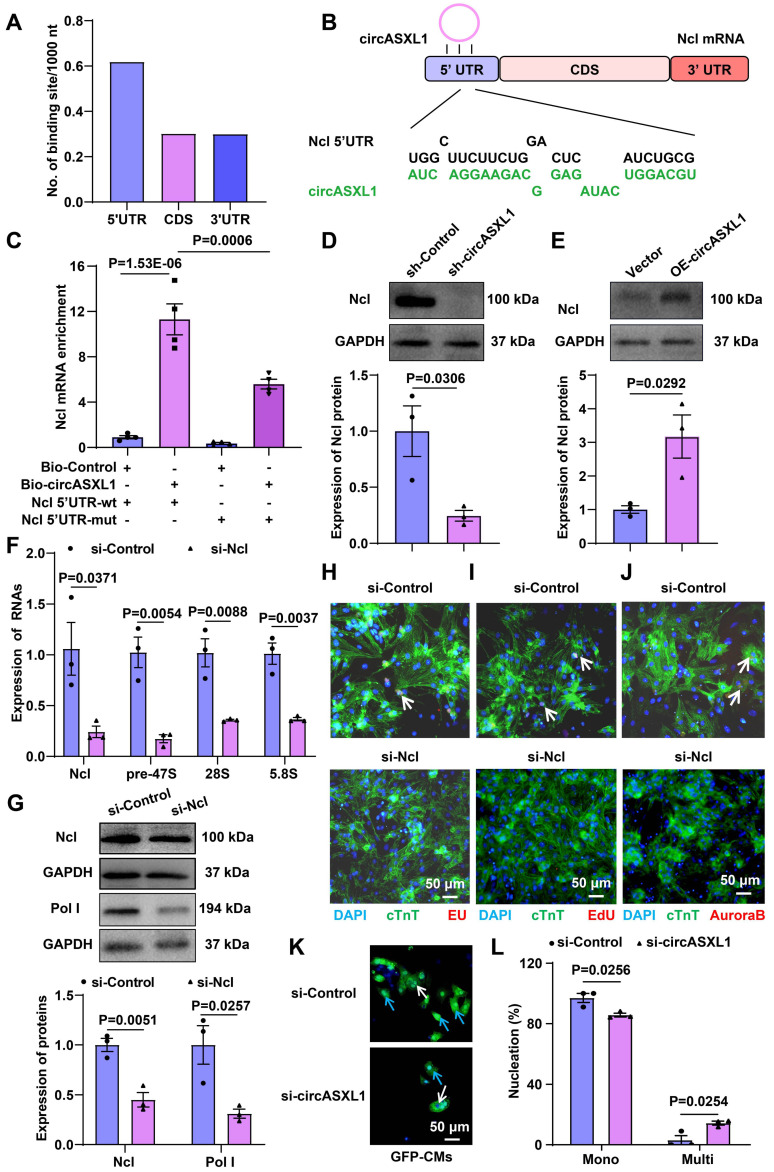
** circASXL1 promotes Ribo-bio by enhancing Ncl expression.** (**A**) The density of circASXL1 binding sites in the designated regions of Ncl mRNA. (**B**) Bioinformatics prediction using the RNAhybrid data base revealed that circASXL1 has a continuous binding site with the 5' UTR of Ncl mRNA. (**C**) The interaction between circASXL1 and Ncl mRNA 5' UTR was confirmed by RNA pulldown assay (n=4). (**D, E**) Western blot analysis of the expression of Ncl protein in CMs transfected with circASXL1 shRNA or overexpression plasmid (n=3). (**F**) RT-qPCR analysis showed that Ncl siRNA treatment led to reduced levels of pre-47S rRNA, 28S, 5.8S transcript (n=3). (**G**) Western blot analysis of the expression of Ncl and Pol Ι protein in CMs transfected with Ncl siRNA (n=3). (**H-J**) rRNA synthesis, proliferation, and cytokinesis of CMs transfected with Ncl siRNA (si-Ncl) or control (si-Control) were determined by EU staining (red), EdU incorporation, and Aurora B staining, respectively (n=5). (**K, L**) The primary CMs from GFP mice were transfected with si-circASXL1 or si-control. Nuclei were identified by DAPI staining. Blue arrow: mononuclear; White arrow: multinucleated (n=3). Data are presented as the mean±SEM.

Additionally, circRNA pull-down assays demonstrated the direct binding of circASXL1 to Ncl (Figure S14A). Further analysis revealed that circASXL1 directly binds to the Ncl mRNA 5'UTR (Figure 7A-7C, Figure S14B), influencing Ribo-bio by promoting the expression of Ncl protein (Figure 7D and 7E). Silencing Ncl in CMs resulted in decreased expression of Ncl and pre-47S rRNA, 28S, 5.8S (Figure 7F and 7G). Silencing Ncl in primary CMs also led to reduced rRNA synthesis (EU incorporation), proliferation (EdU incorporation), and cytokinesis (Aurora B staining) (Figure 7H-7J). Moreover, the number of mononuclear CMs from GFP mice was diminished by si-circASXL1 (Figure 7K and 7L). These data collectively indicate that circASXL1 promotes cytokinesis via Ncl.

### Exo circASXL1 drives MI repair by augmenting ribo-bio

**Figure 8:**
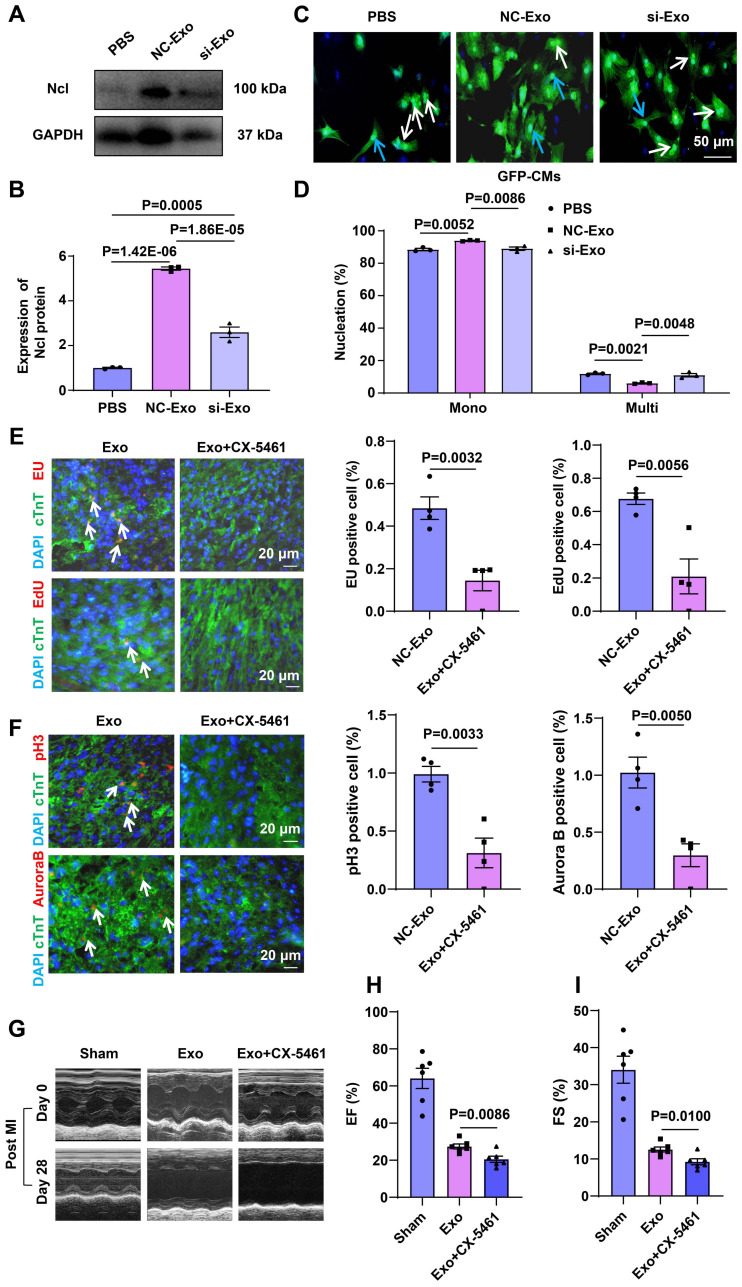
** Exosome-derived circASXL1 promotes cytokinesis by enhancing Ribo-bio.** (**A, B**) Western blot analysis of the expression of Ncl protein in hearts treated with NC-Exos or si-Exo (n=3). (**C, D**) NC-Exo treatment resulted in increased mononuclear CMs derived from GFP mice. Nuclei were identified by DAPI staining. Blue arrow: mononuclear; White arrow: multinucleated (n=3). (**E, F**) Immunofluorescence staining of EU, EdU, pH3, Aurora B showed that the enhanced rRNA synthesis, proliferation, and cytokinesis exerted by exosomes was attenuated by Ribo-bio inhibitor CX-5461 (n=4). (**G-I**) Representative echocardiography and cardiac function of different treatment groups (n=6). Data are presented as the mean±SEM.

To ascertain whether circASXL1 in UMSC-Exo triggers ribo-bio following MI, we silenced circASXL1 in UMSC, yielding exosomes deficient in circASXL1 (si-Exo). Mice were divided into phosphate-buffered saline (PBS), control exosomes (NC-Exo), and si-Exo groups. NC-Exo treatment resulted in increased Ncl protein expression, a response blunted by si-Exo (Figure 8A and 8B). NC-Exo treatment also led to an increased number of mononuclear CMs from GFP mice, a response attenuated by si-Exo (Figure 8C and 8D). The rRNA synthesis (EU incorporation), proliferation (EdU incorporation), and cytokinesis (Aurora B staining) were diminished in mice treated with Ribo-bio inhibitor CX-5461 (Figure 8E and 8F). The beneficial effects of Exo on cardiac function (Figure 8G-8I, Table S4) and fibrosis reduction were mitigated by CX-5461 (Figure S15A-15C). *In vitro*, CX-5461 hampered Ribo-bio (Figure S16A-16E). These findings suggest that exosome-derived circASXL1 fosters cytokinesis by enhancing Ribo-bio.

## Discussion

**Figure 9:**
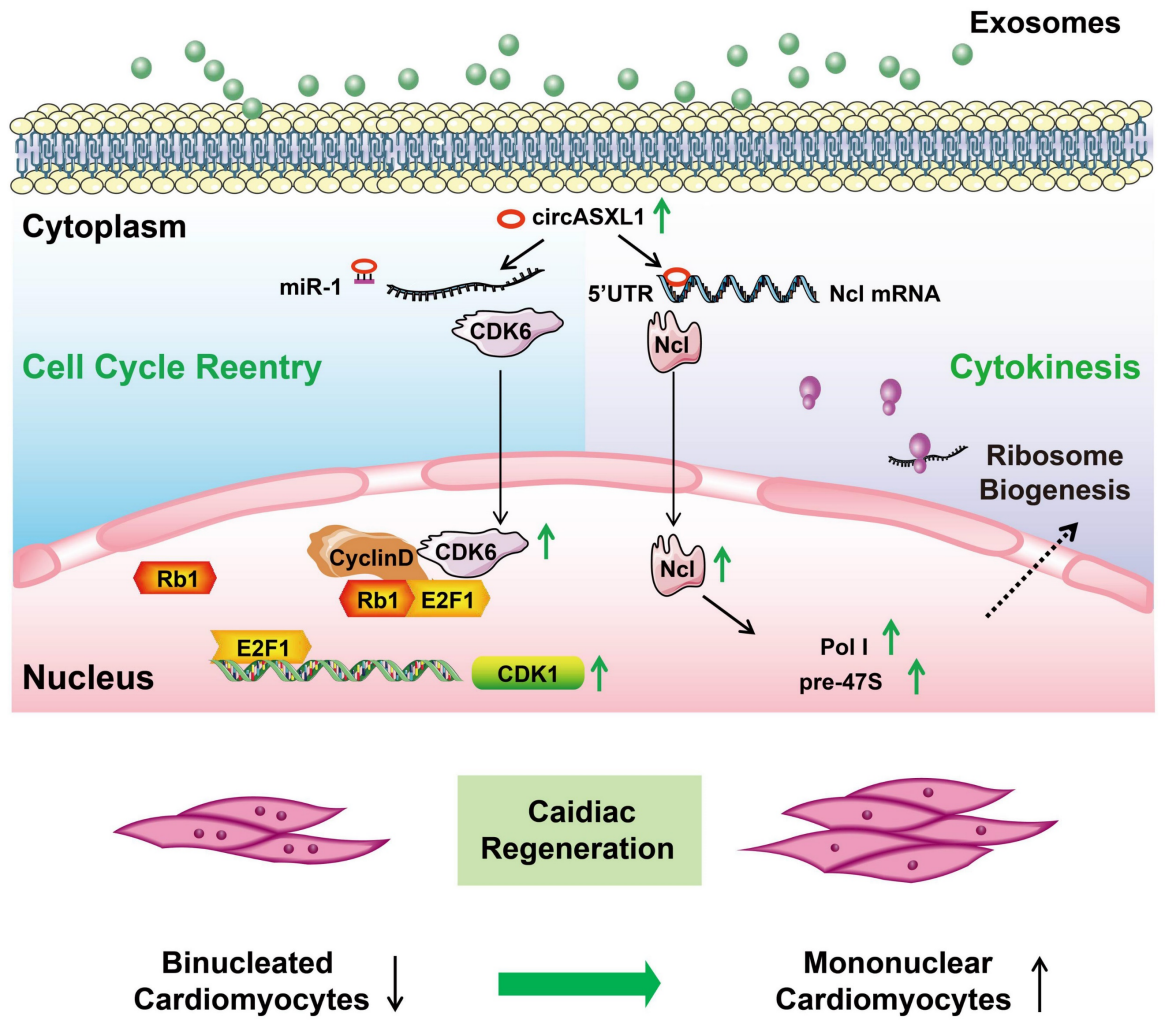
** Proposed mechanisms of exosomal circASXL1 in cardiac repair.** The series signaling of circASXL1/CDK6/Rb1/cell-cycle reentry and circASXL1/Ncl/Ribo-bio/cytokinesis plays a crucial role in cardiac repair.

In this investigation, employing discovery-driven methodologies such as circRNA/mRNA/protein sequencing, Ribo-Halo, Ribo-disome, circRNA pull-down assays, and utilizing a cardiac-specific Rb1-knockout mouse model, we unveiled a pivotal role of the circASXL1/Rb1 signaling pathway in cell-cycle reentry and the circASXL1/Ncl/Ribo-bio signaling axis in cytokinesis. Our findings underscore the significant contribution of circ-ASXL1 derived from UMSC-Exo in MI repair, orchestrating crucial signaling network in CM cell-cycle reentry and cytokinesis (Figure 9).

A previous investigation revealed that tumor suppressor Rb1 hinders proliferation by impeding cell-cycle reentry 30, as demonstrated in our prior study showing the prolonged enhancement of skeletal muscle repair following ischemic injury upon Rb1 gene knockout 31. In the context of MI, our current study affirmed that suppressing Rb1 promotes cell-cycle reentry and proliferation; however, the *in vivo* benefits of Rb1 silencing are transient. Intriguingly, our study unveils that combining Rb1 silencing with the activation of Ribo-bio can facilitate enduring cardiac repair.

In this study, we observed a reduction in Ribo-bio within infarcted myocardium, and enhancing cardiac repair by stimulating Ribo-bio through Ncl activation. Ncl plays a crucial role in pre-rRNA processing and rRNA maturation. Our results underscore the significance of Ribo-bio as a pivotal process for cytokinesis, shedding light on the earlier observation that Ncl protein plays an important role in maintaining cardiac function 32. Importantly, our study establishes Ncl as a regulator of Ribo-bio, and demonstrates that UMSC-derived circASXL1 promotes the expression of Ncl. These findings carry clinical significance in the realm of cardiac regeneration.

Furthermore, mRNAs encompass not only the coding sequence (CDS) but also include 5' untranslated regions (5' UTR) and 3' UTR. These UTRs play pivotal roles in regulating gene expression, pre-mRNA splicing, maintaining mRNA stability, and initiating translation 33. Notably, it has been observed that the 3' UTR of Ckip-1 independently inhibits cardiac hypertrophy, irrespective of its associated protein 34. However, the significance of the UTR in Ncl mRNA remains elusive. Our discovery reveals the binding affinity of circASXL1 to Ncl mRNA, with a detailed analysis highlighting the highest binding density in the 5' UTR compared to the 3' UTR and CDS. This suggests that circASXL1 may exert its functions by interacting with the 5' UTR of Ncl mRNA. The exploration of circRNA's regulatory impact on target mRNA UTR introduces a novel avenue in circRNA research. Future investigations are warranted to elucidate whether Ncl 5' UTR independently promotes Ribo-bio.

A microarray analysis revealed a significant downregulation of circASXL1 following MI 35, yet the role of circASXL1 in cardiac regeneration remained unexplored. In this study, we elucidated that circASXL1 released by UMSC-Exo plays a crucial role in promoting cardiac repair by modulating the miR-1/CDK6/Rb1 signaling network. MiR-1 has been implicated in the development of cardiovascular diseases 36, and its inhibition has been associated with improved cardiac function by enhancing insulin-like growth factor-1 (IGF-1) signaling. CDK6, a member of the CDK family, induces cell-cycle progression by releasing the transcription factor E2F through Rb1 inactivation 37. Our findings revealed CDK6 as a novel downstream target of miR-1, alongside IGF-1. The downregulation of miR-1 signaling by circASXL1 enhances cell-cycle reentry/proliferation by increasing CDK6 expression and inactivating Rb1. Significantly, circASXL1 emerges as a novel upstream regulator of miR-1. The enriched presence of circASXL1 in UMSC-Exo enhances its efficacy in treating MI compared to a miR-1 inhibitor, as it can be delivered to injury sites without degradation.

E2F1 typically forms a complex with Rb1, and the modulation of Rb1 leads to the release of E2F1, which is important in cell cycle regulation by influencing the expression of target genes. CDK1 stands out as one of the target genes impacted by E2F1, particularly in the regulation of the G2/M phase 38. While this study did not extensively examine the regulatory role of CDK1 in CMs proliferation, our findings indicated that silent-Rb1 heightened the G2/M phase percentage of CMs.

Earlier investigations have highlighted the pivotal role of Ribo-bio in cytokinesis, with Ncl playing a regulatory role in Ribo-bio and influencing cell growth 39. Building upon this knowledge, our study demonstrates that the circASXL1/Ncl/Ribo-bio signaling network enhances cardiac regeneration by promoting cytokinesis and diminishing the occurrence of dysfunctional binucleated CMs. Remarkably, we present the first evidence that UMSC-Exo contribute to MI repair by concurrently stimulating CM cell-cycle reentry/proliferation and cytokinesis.

In summary, we have uncovered novel mechanisms orchestrating cell-cycle reentry and Ribo-bio through the circASXL1/Rb1 and circASXL1/Ncl axes, respectively. Significantly, we have demonstrated that UMSC-Exo derived circASXL1 contributes to MI repair by concurrently stimulating CMs cell-cycle reentry/proliferation and cytokinesis. These findings advance our understanding of the molecular underpinnings of cardiac repair and offer potential avenues for developing innovative therapeutic strategies targeting the circASXL1 signaling network in MI.

## Methods

### Harvesting and identification of UMSC exosomes

Exosomes were obtained and characterized following our previously reported procedures 18. UMSCs, purchased from Jiangsu Heze Biotechnology Co., Ltd, China, were cultured in α-minimum essential medium containing 10% fetal bovine serum (FBS). The FBS was pre-cleared of bovine-derived exosomes by centrifugation at 100,000 g. After 48 hours of culture, exosomes were isolated from the supernatant using a total exosome isolation kit (Life Technology, San Francisco, CA, USA), providing a substantial yield of purified exosomes. The UMSC culture medium was initially centrifuged at 2,000 g for 30 min to eliminate dead cells and debris. Subsequently, the supernatant was mixed with 0.5 volumes of the Total Exosome Isolation reagent, incubated overnight at 4 °C, and centrifuged at 10,000 g for 1 h at 4 °C. The resulting pellet was re-suspended in PBS and stored at -80 °C. The protein concentration of exosomes was quantified using a BCA protein assay kit (Takara, Japan). Due to the small size of exosomes, which cannot be directly detected by flow cytometry, they were pre-bound to aldehyde/sulfate latex beads (4 μm; Molecular Probes; Invitrogen, Waltham, MA, USA) to enhance the signal, followed by incubation with a fluorescein isothiocyanate-conjugated antibody targeting the exosome surface marker CD63. Flow cytometry analysis was then performed, as previously described 18. The expression of CD63 was also assessed using Western blot.

### Generation of cardiac-specific Rb1-knockout (Rb1-KO) mice

To create conditional Rb1-cKO mice (Rb1^flox/Wt^), we designed a construct for the conditional disruption of the Rb1 gene, featuring two loxP sites flanking the third exon of the Rb1 gene. The vector targeting ES cells comprised a 4.0-kb 5' homology arm, a 0.7-kb flox region, PGK-Neo-polyA, a 4.0-kb 3' homology arm, and an MC1-TK-polyA negative selection marker. Following linearization of the vector, ES cells underwent electrical transfection. Positive clones were selected through G418 screening and confirmed by long-fragment PCR. Clones displaying accurate homologous recombination were expanded and injected into blastocysts of C57BL/6J mice to generate chimeric mice (Rb1^flox/Wt^). The generation of Rb1^flox/Wt^ mice was performed by the Shanghai Model Organisms Center, Inc.

To produce cardiac-specific Rb1-cKO mice, α-MHC^MerCreMer^ mice were bred with Rb1^flox/Wt^ mice. The initial cross involved mating α-MHC^MerCreMer^ mice with Rb1^flox/Wt^ mice to yield α-MHC^MerCreMer^Rb1^flox/Wt^ intermediate mice. Subsequently, these heterozygous mice were further bred to obtain cardiac-specific Rb1-KO mice. Genotyping was performed using specific primers:

Cre F: 5'-CAGCATTGCTGTCACTTGGTC-3';

Cre R: 5'-ATTTGCCTGCATTACCGGTCG-3';

loxp F: 5'-GCCCTTGGAGCTGGAGTTAGA-3';

loxp R: 5'-AATGCCTGGGTCAAGTGTCAATCA-3'.

All animal procedures were conducted in accordance with the Guidelines or the Care and Use of Laboratory Animals and were approved by the Institutional Animal Care and Use Committee at Soochow University (Suzhou, China).

### GFP transgenic mice

The transgenic β-actin-GFP mice were purchased from Cyagen Bioscience. All animal care and experimental procedures strictly adhered to approved protocols and animal welfare regulations set by the Animal Care and Use Committee at Soochow University. All surgical interventions were conducted under isoflurane anesthesia, and every effort was made to minimize the mice's discomfort and suffering.

### Isolation and culture of nenonatal cardiomyocytes

Cardiomyocytes (CMs) were isolated from 1-day-old transgenic β-actin-GFP pups or C57BL mice using a previously established method 40. In brief, following euthanasia, ventricles from the hearts extracted from the pups were minced, and the CMs were dissociated using trypsin (Beyotime, C0201). The cells were then suspended in DMEM (GIBCO) supplemented with 10% FBS (Biological Industries), and initially plated in culture dishes for 60 minutes to allow fast-adherent cells (fibroblasts) to attach. Non-adherent cells (CMs) were subsequently collected, plated in culture dishes, and selected based on their attachment (CMs).

### circRNA pull-down

Following transfecction of 293T cells with either the Ncl mRNA 5'UTR wild-type plasmid or the Ncl mRNA 5'UTR mutant plasmid, cell lysates were co-incubated with circASXL1 probes. DNA oligo probes, designed by RiboBio Biotechnology, contain BiotinTEG at 5' end and target the back-splice junction of circASXL1. Subsequent to transfection with the Ncl mRNA 5' UTR plasmid, 293T cells were collected, crosslinked with 1% glutaraldehyde at room temperature for 10 min, and then reaction was quenched with 1.25 M glycine. Crosslinked cells underwent lysis and sonication. Chromatin was subjected to hybridization with biotinylated circASXL1 probes at 37 °C for 4 hours. Streptavidin-conjugated beads (Invitrogen, Cat#65001) were introduced and incubated for 30 min. The precipitated RNA was subsequently identified and quantified through qPCR.

### MI induction and implantation of UMSC exosomes

MI was induced in male C57BL/6 mice (aged 8-10 weeks), following previously reported methods 18. In brief, mice were anesthetized through spontaneous inhalation and maintained under general anesthesia with 1-2% isoflurane. Mechanical ventilation was administered using a rodent ventilator connected to an endotracheal tube. A chest retractor was carefully positioned in the fourth intercostal space. Following exposure of the left ventricle, the left anterior descending (LAD) coronary artery was ligated using an 8-0 nylon suture. The successful induction of MI was confirmed by an immediate color change in the infarcted area. Subsequent to LAD ligation, PBS (30 μL) and exosomes (100 μg/30 μL) were injected into three different sites along the infarct border.

### Cell culture and the loss/gain-of-function approach

H9C2 cells were cultured in Dulbecco's modified Eagle's medium (DMEM) supplemented with 10% FBS at 37 °C in 5% CO_2_. The miR-1 mimic, miR-1 inhibitor, and small-interfering RNA (si-circASXL1) were synthesized by Ribobio (Guangzhou, China). Transfection was performed using Lipofectamine 2000 (Invitrogen, Carlsbad, CA, USA) following the manufacturer's instructions. For experiments involving Exo-si-circASXL1, the cells were transfected with si-circASXL1 targeting circASXL1 (CACGCTCAAGGTATTAGAA) for 48 hours, and then exosomes were isolated as described previously 19. Additionally, for experiments involving sh-circASXL1, the cells were transfected with a sh-circASXL1 plasmid targeting circASXL1 (CTCAAGGTGTTAGAAAACT). An siRNA designed to target the coding region of Rb1 mRNA (GTCAAGGGCTTACCATACT) was synthesized by Ribobio (Guangzhou, China). Negative control experiments utilized scrambled siRNAs that do not induce the degradation of any known cellular mRNA. H9C2 cells (3 × 10^5^) were incubated with the siRNAs (100 nM) prior to experiments.

### Ribo-bio EU assay

To evaluate ribosome biogenesis (Ribo-bio), an EU Assay kit (RiboBio, Guangzhou, China) was employed. Following various treatments, H9C2 cells were incubated in fresh medium containing 10 μM EU for 2 hours. Subsequently, the cells were washed with PBS, fixed in 4% paraformaldehyde for 30 min, and treated with 0.5% Triton X-100 for 10 min. Nuclei were stained with DAPI for 15 min. The percentage of cells incorporating EU was determined through fluorescence microscopy.

### Ribo-bio AgNOR staining

Silver staining of NORs in control cells and cells treated with circASXL1 siRNA or Ncl siRNA was conducted following established AgNOR procedures 41. In brief, after fixation, H9C2 cardiomyocytes were subjected to staining with a freshly prepared AgNOR staining solution for 30 minutes. Following staining, H9C2 were rinsed twice in ddH_2_O and mounted for bright field microscopy using a camera for image capture.

### Ribo-HaloTag assay

The RPS3-HaloTag7 and RPL29-HaloTag7 plasmids were constructed by Fenghui Biotechnology (Changsha, China). Ribosomes were labeled using a previously established method 29. In brief, 293T cells were seeded onto 24 well plates and transfected with the RPS3-HaloTag7 and RPL29-HaloTag7 plasmids to generate Ribo-Halo cells. TMR-Halo ligand at a concentration of 100 nM was added and incubated for 1 h to label the pre-existing ribosomes. Details about the ligands used in this study are provided in Table S5. The cells were incubated with 1 ml of fresh DMEM for 5 min in the dark to eliminate the remaining free TMR-ligand. Subsequently, the cells were transfected with circRNA shRNA or circRNA overexpression plasmid, and treated with 50 nM R110 Halo ligand for 24 h to label the newly synthesized ribosomes. After treatment, the cells were collected, and nuclei were stained with DAPI for 15 min. The proportion of Halo-cells was ultimately determined using fluorescence microscopy.

### Ribo-disome assay

Ribosome was isolated on a sucrose gradient employing a previously documented method 42. Cells were cultured until reaching 70-80% confluency and then treated with actidione for 10 min. Subsequent to treatment, cells were rinsed with PBS and lysed in lysis buffer. The digested lysates were passed through 10%-35% sucrose gradients using Beckmann Coulter at 40,000 rpm for 4 °C for 2 h. The absorbance at 260 nm was measured.

### Statistical analysis

Data were analyzed using GraphPad Prism 8, and are presented as the mean±SEM. Two-tailed t-tests were used to determine the significance of differences between two groups. Comparisons between multiple groups were assessed by ANOVA with Tukey's multiple comparisons test. P <0.05 was considered statistically significant.

## Supplementary Material

Supplementary methods, figures and tables.
