# Supplementary material for: The orchestration of cell-cycle reentry and ribosome biogenesis network is critical for cardiac repair
Source: Theranostics. 2024 Jun 24;14(10):3927–44. doi: 10.7150/thno.96460 (PMC11234283; doi:10.7150/thno.96460)
Supplement: Supplementary file 1 — Supplementary methods, figures and tables. [file thnov14p3927s1.pdf]

Supplementary Materials for

The orchestration of cell-cycle reentry and ribosome biogenesis network is critical for cardiac repair

Short title: Ribosome biogenesis is critical for cardiac repair

Yanli Wang[1#], Junchu Tu[1#], Weiliang Wu[1#], Yan Xu[2#], Yujie Li[1], Xiangbin Pan[3], Bin Liu[4], Tonggan Lu[1], Qingfang Han[1], Huiling Zhang[1], Lijuan Jiao[1], Yu Zhang[1], Xi-Yong Yu[5], Zhenya Shen[1]*, Yangxin Li[1]*

[#]These authors contributed equally.

*Correspondence to:

Address correspondence to: Yangxin Li, PhD, FAHA

Institute for Cardiovascular Science and Department of Cardiovascular Surgery,

First Affiliated Hospital of Soochow University, Suzhou, Jiangsu 215123, P. R. China

Tel: 86-512-67781962, or Fax: 86-512-67780100,

Email: yangxin_li@yahoo.com

The file includes:

Supplementary Methods

Figure S1-S16

Tables S1-S7

Supplementary methods

RNA extraction and real-time PCR

RNA extraction was carried out utilizing TRIzol reagent (Takara). Subsequently, total RNA was reverse-transcribed using a PrimeScript™ RT reagent kit (Takara). Real-time PCR was conducted using a SYBR Premix Ex Taq™ kit (TliRNaseH Plus) (Takara) and the ABI (Foster City, CA, USA) StepOnePlus Real-Time PCR System. The primer sequences are presented in Table S6.

For circRNA and miRNA quantification, GAPDH and U6 were utilized as reference genes, respectively. The primers for miR-1 and U6 were obtained from RiboBio (Guangzhou, China). The circRNA/mRNA reaction conditions were as follows: pre-denaturation at 95 °C for 30 s, followed by followed by 40 cycles denaturation at 95 °C for 5 s, and annealing at 60 °C for 30 s. The reaction conditions for miRNA qPCR were pre-denaturation at 95 °C, 10 min, followed by 40 cycles denaturation at 95 °C for 2 s, and annealing at 60 °C for 30 s. Each gene was analyzed in triplicate wells, and the gene expression was calculated by the $2^{-\Delta\Delta Ct}$ method, with each experiment repeated three times.

Western blot

Cells were rinsed with PBS and lysed in lysis buffer on ice for 30 min. Following centrifugation at 12,000 g for 10 min, the protein concentration in the supernatant was determined using a BCA kit. The protein extracts were separated by polyacrylamide gel electrophoresis (12%) and subsequently transferred onto polyvinylidene difluoride membranes. The primary antibodies against cTnT (catalog #ab8295), Aurora B (catalog #ab2254), pH3 (catalog #ab47297) and Rb1 (catalog #ab181616) were from Abcam (Cambridge, UK). The antibodies against Ncl (catalog #14574S), CDK6 (catalog #3136S), RPS3 (catalog #9538S) were from Cell Signaling Technology

(Boston, MA, USA). Primary antibodies against RNA Pol I (catalog #sc-48385), CD63 (catalog #sc-5275) , TSG101 (catalog #sc-7964) and GAPDH (catalog # sc-32233) were from Santa Cruz Biotechnology (Dallas, TX, USA). Primary antibody against CDKN2a (catalog #32050) was from Signalway Antibody (Maryland, USA). Primary antibody against RPL29 (catalog #15799-1-AP) was from Proteintech Group (Chicago, IL, USA). Details regarding the quantity and application of the antibodies used in this study are provided in Table S7. The antibodies were appropriately diluted in NCM Universal Antibody Diluent (catalog # WB100D). Protein signals were visualized using an ECL chemiluminescence kit (catalog # P10200) from New Cell & Molecular Biotech Co., Ltd (Suzhou, China), and the luminescence was captured with a BioRad luminescent imaging system.

Echocardiography

Transthoracic echocardiography was conducted using Visual Sonics Vevo 2100 system equipped with a 40-MHz probe. Hearts were visualized in a 2D long-axis view at the point of the maximum left ventricle (LV) diameter in lightly anesthetized animals. This view was used to position the M-mode cursor perpendicular to the LV anterior and posterior walls. Cardiac function was assessed under light anesthesia (1.0% inhaled isoflurane) at 7 or 28 days post MI using a 21-MHz transducer (Visual Sonics). The left ventricular ejection fraction was determined according to established methods [19]. All procedures and analyses were carried out by a researcher blinded to the treatment groups.

Dual-luciferase reporter assay

The psiCHECK^{TM-2} vector containing the firefly luciferase reporter gene was obtained from Promega. The circASXL1 sequence was cloned into the vector, resulting in the circASXL1-WT construct. To introduce a mutation in the miR-1 binding site, a

circASXL1 sequence with the mutation was synthesized by GENEWIZ Biological Technology Co., Ltd (Suzhou, China), cloned into the vector, and designated as circASXL1-Mut. Sequencing was performed to confirm the integrity of the constructed vectors. The primer sequences used for construction were as follows: for the WT, forward: 5'-TCGACTCAATGCTATGCTACATTCCA-3', reverse: 5'-GGCCTGGAATGTAGCATAGCATTGAG-3'; and for the Mut, forward: 5'-TCGACACATAGGAATGCATGTAAGGT-3', reverse: 5'-GGCCACCTTACATGCATTCCTATGTG-3'. HEK293T cells were seeded in 96-well plates (5×10^3 cells per well) and cultured for 24 h before transfection. The cells were co-transfected with a mixture of 50 ng reporter plasmid (circASXL1-miR-1-WT or circASXL1-miR-1-Mut), with 200 nM miR-1 mimic from RiboBio. After 48 h, the luciferase activity was analyzed using a dual luciferase reporter assay system (Promega, USA) per the manufacturer's instructions.

EdU proliferation assay

Cell proliferation was evaluated using an EdU Cell Proliferation Assay kit (RiboBio, Guangzhou, China). Following various treatments, H9C2 cells were cultured in fresh medium containing 10 μM EdU for 2 h. Subsequently, the cells were washed with PBS, fixed in 4% paraformaldehyde for 30 min, and permeabilized with 0.5% Triton X-100 for 10 min. Nuclei was stained with DAPI for 15 min. The proportion of cells incorporating EdU was then determined by fluorescence microscopy.

mRNA sequencing and analysis

Total RNA was isolated from myocardium tissues using TRIzol reagent (Invitrogen). Subsequently, cDNA was synthesized from ribosome-depleted RNA samples using random hexamer primers. The whole transcriptome sequencing data obtained from the HiseqTM Sequencer underwent filtering to eliminate adaptor sequences, reads

with >5% ambiguous bases, and low-quality reads containing >20% of bases with quality <20. The resulting data were aligned to the mouse genome using HISAT2 and gene counts of mRNA were calculated using HTSeq. All RNA-seq and bioinformatics analyses were conducted at Novel Bio Ltd (Shanghai, China).

Sample preparation for proteomics sequencing

The tissues were pulverized in liquid nitrogen, and then lysed in lysis buffer containing 7 M urea, 4% SDS, and a protease inhibitor cocktail (Roche Ltd. Basel, Switzerland), followed by sonication on ice. Afterward, the samples were centrifuged at 13,000 rpm for 10 min at 4 °C to remove insoluble particles. The resulting supernatant was collected, and the protein concentration was determined using the BCA protein assay. Aliquots of the supernatant containing 100 μg protein were transferred to new tubes, and the final volume was adjusted to 100 μL with 100 mM TEAB (triethylammonium bicarbonate). The samples were incubated with 5 μL DTT (200 mM) at 55 °C for 1 h to break the disulfide bond. Subsequently, 5 μL of 375 mM iodoacetamide was added and incubated for 30 min to prevent the potential re-formation of the disulfide bond. The proteins were then precipitated with ice-cold acetone, dissolved in 100 μL TEAB, digested with sequence-grade modified trypsin (Promega, Madison, WI), and the resulting digested peptide mixture was labeled using chemicals from the iTRAQ reagent kit. The labeled samples were combined, desalted on a C18 SPE column (Sep-Pak C18, Waters, Milford, MA), and dried in a vacuum.

High pH reverse-phase separation

The peptide mixture was dissolved in a buffer containing 10 mM ammonium formate in water, adjusted to pH 10.0, and subsequently fractionated by linear gradient high pH separation using an Aquity UPLC system (Waters Corp., Milford, MA). The

column flow rate was maintained at 250 μL/min and column temperature was set at 45°C. Twelve fractions were collected, with each fraction being dried in a vacuum concentrator for the subsequent steps.

Low pH Nano-HPLC-MS/MS analysis

The fractions were re-suspended with 40 μL solvent C (water with 0.1% formic acid D: ACN with 0.1% formic acid), then subjected to separation by nanoLC, and analyzed by on-line electrospray tandem mass spectrometry. The analysis employed an EASY-nLC 1000 system (Thermo Fisher Scientific, Waltham, MA) coupled with an Orbitrap Fusion Mass Spectrometer (Thermo Fisher Scientific, San Jose, CA), equipped with an online nano-electrospray ion source. Loading of the samples (4 μL) onto the trap column occurred at a flow rate of 10 μL/min for 3 min, followed by separation on the analytical column with a linear gradient from 5% D to 30% D in 110 min.

Quantitative aata analysis

All proteomics sequencing and associated bioinformatics analyses were conducted at Biotree Biotech Co., Ltd (Shanghai, China). In brief, the percolator algorithm was employed to maintain peptide-level false-discovery rates below 1%. Protein quantification relied solely on unique peptides. Proteins contained at least two unique peptides, and the method of normalization to the median was used to correct experimental bias; the minimum number of proteins was set to 1000.

Supplementary figures

Figure S1

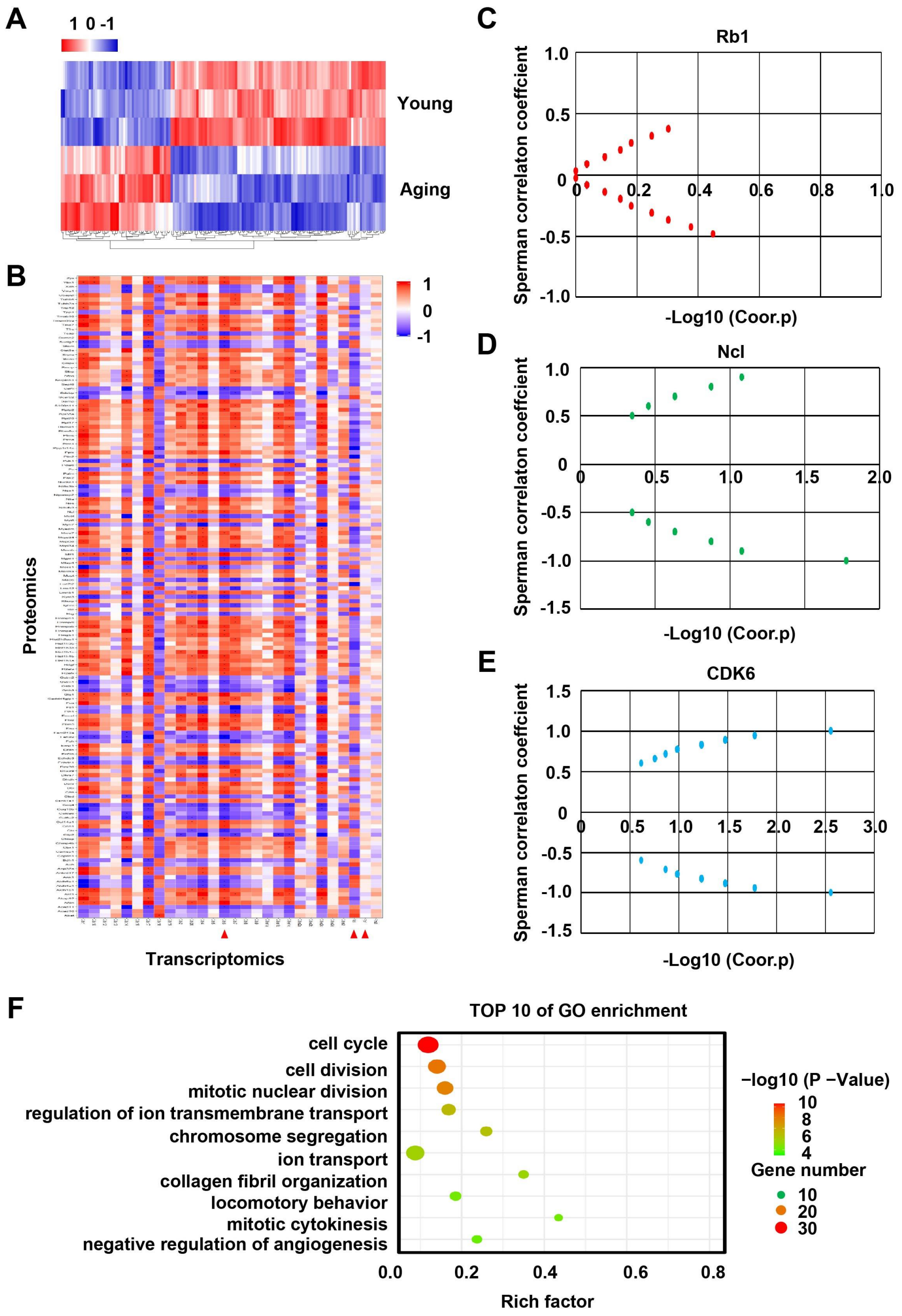

Figure S1. Correlation between mRNA and protein-sequencing in aging and young heart. (**A**) Heatmap illustrating protein-sequencing data from the hearts of aging and young mice (blue, downregulated; red, upregulated). (**B**) Protein-mRNA correlation was analyzed by Spearman's correlation coefficients using normalized

scores (z-scores). (**C**) Spearman's correlation analysis demonstrating the correlation between increased mRNA expression of Rb1 and the protein expression changes in aging vs. young mice. (**D, E**) Spearman's correlation analysis indicating the correlation between decreased mRNA expression of Ncl and CDK6 with the protein expression changes in aging vs. young mice. (**F**) ceRNA-GO analysis in aging mice compared with young mice.

Figure S2

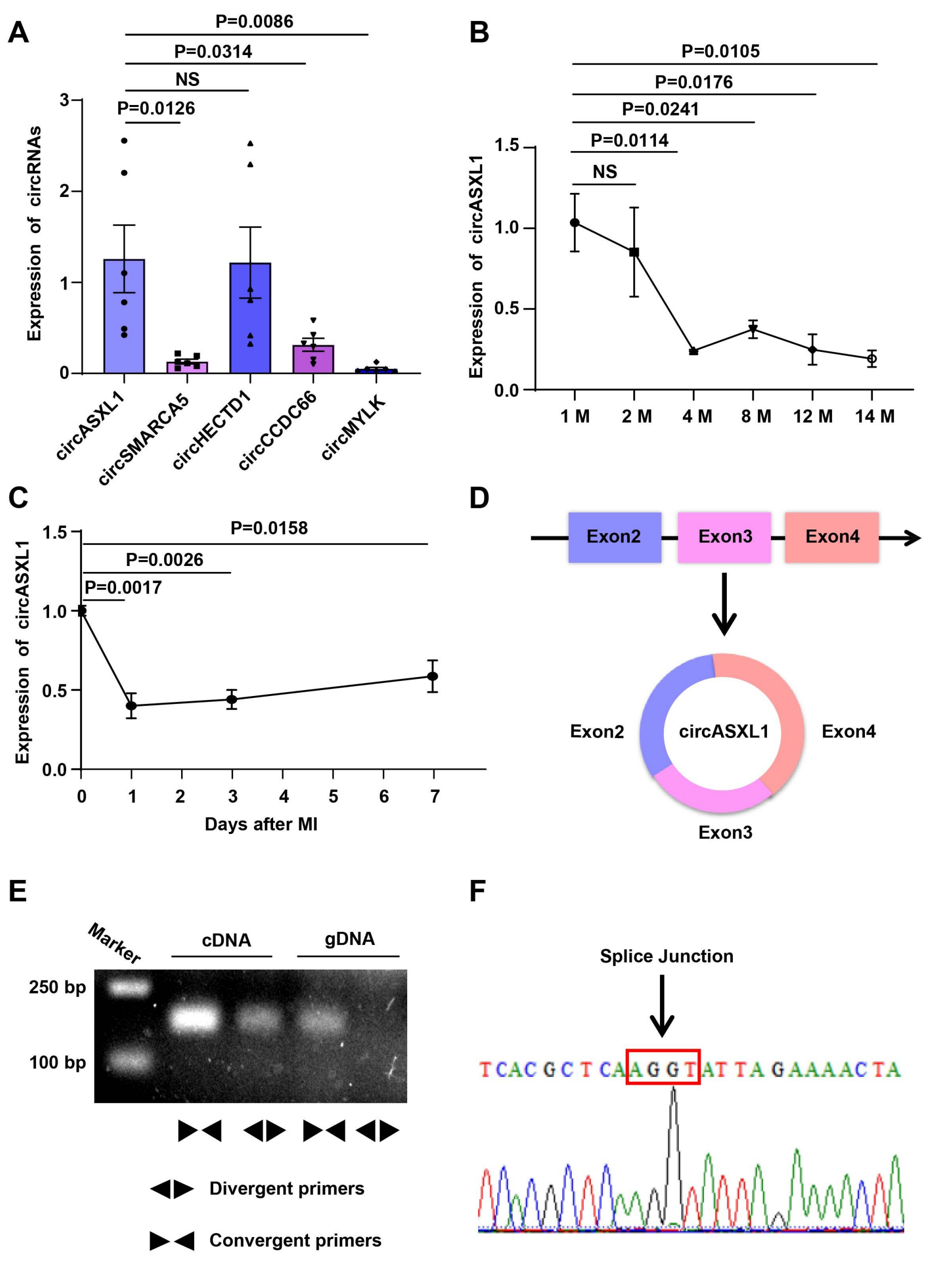

Figure S2. circASXL1 expression in ischemic heart injury. (**A**) RT-qPCR analysis of the expression of circRNAs in CMs (n=6). (**B**) RT-qPCR analysis of the expression of circASXL1 in the young and aging heart (n=3). (**C**) RT-qPCR analysis of circASXL1 expression at different time points after ischemic injury (n=3). (**D**) Schematic illustration of circASXL1 formation. (**E, F**) Sanger sequencing analysis of the splice junction of circASXL1. Data are presented as the mean ± SEM.

Figure S3

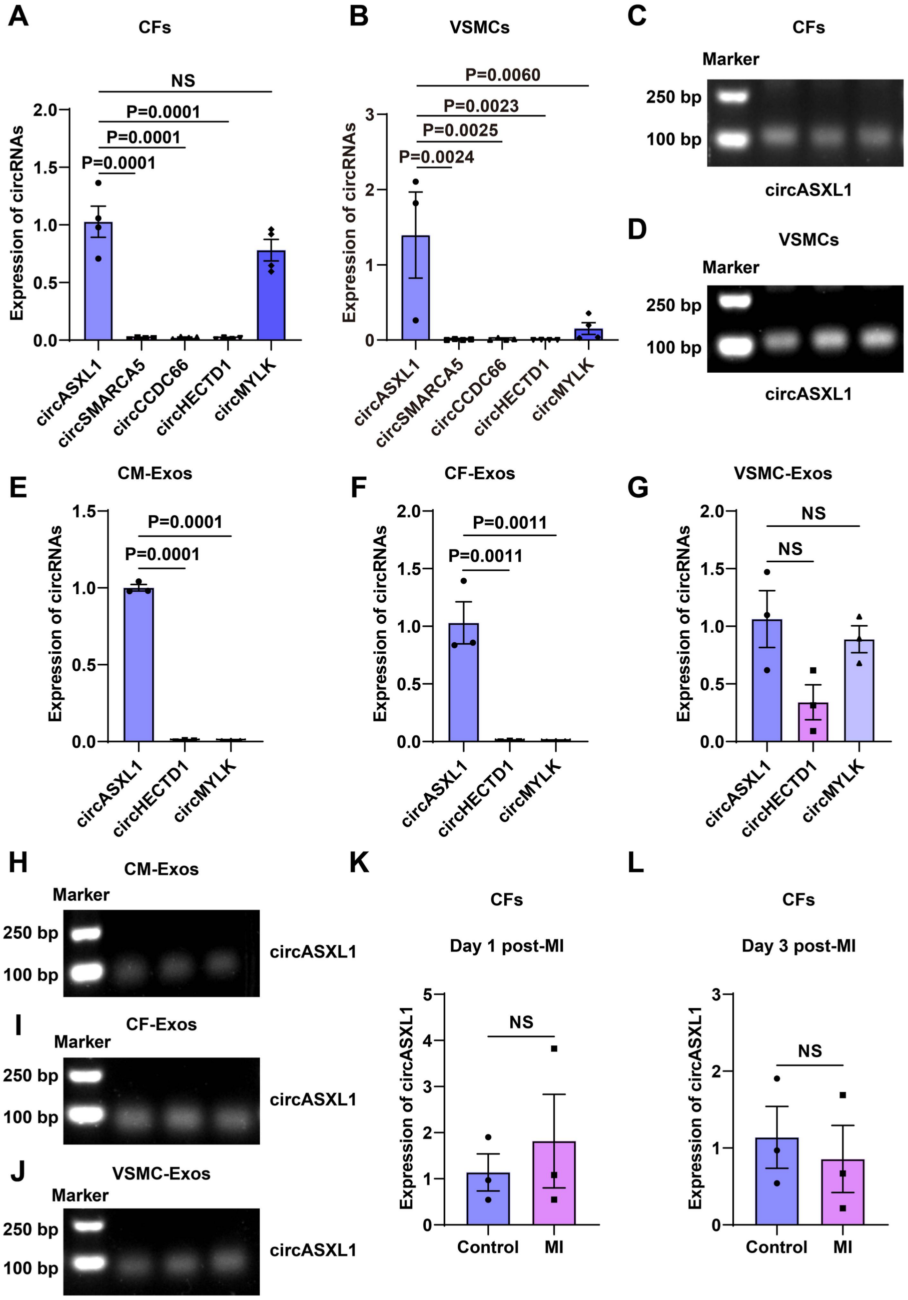

Figure S3. circASXL1 expressed in other cardiac cell and exosomes derived from these cell. (**A-D**) RT-qPCR analysis of the expression of circASXL1 in CFs and VSMCs (n=4). (**E-J**) RT-qPCR analysis of the circASXL1 expression in CMs, CFs and VSMCs exosomes (n=3). (**K, L**) RT-qPCR analysis of the expression of circASXL1 in CFs at different time points after MI (n=3). Data are presented as the mean±SEM.

Figure S4

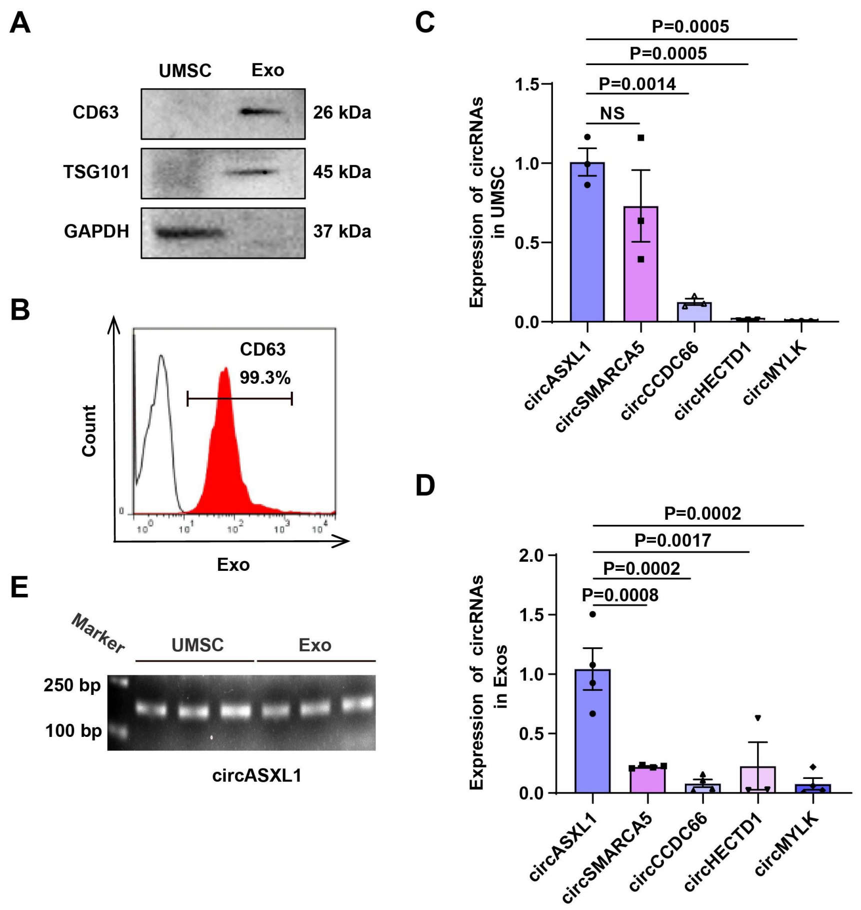

Figure S4. circASXL1 is highly expressed in UMSC-Exo. (**A, B**) Expression of the exosome markers CD63 or TSG101 analyzed by Western blot and flow cytometry (n=3). (**C**) RT-qPCR analysis of the expression of circRNAs in UMSC (n=3). (**D**) RT-qPCR analysis of the expression of circRNAs in UMSC-Exo (n=4). (**E**) Expression levels of circASXL1 in UMSC and UMSC-Exo (n=3). Data are presented as the mean ± SEM.

Figure S5

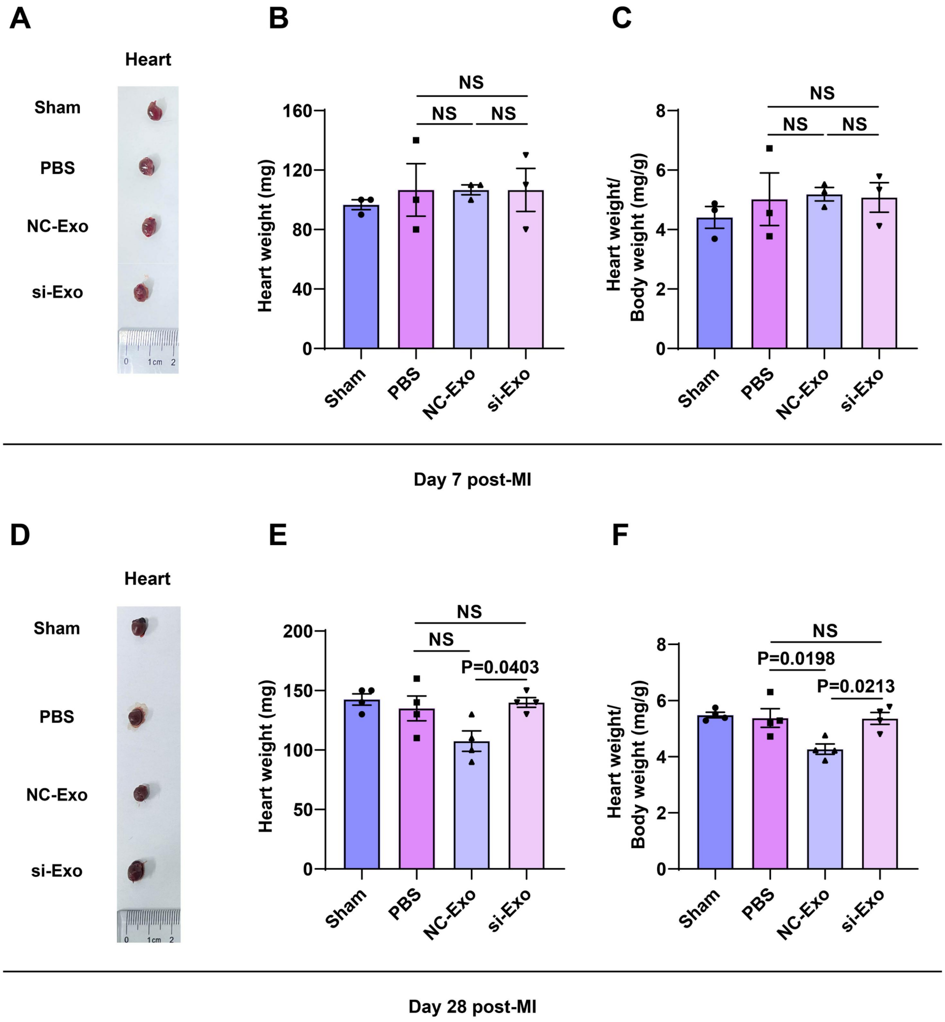

Figure S5. UMSC-Exo–derived circASXL1 can attenuate cardiac hypertrophy. (**A**) Image of cardiac shape across different treatment groups at day 7 post-MI (n=3). (**B**) Heart weight in different groups at day 7 post-MI (n=3). (**C**) Heart weight-to-body weight ratio at day 7 post-MI (n=3). (**D**) Image of cardiac shape across the treatment groups at day 28 post-MI (n=4). (**E**) Heart weight at day 28 post-MI (n=4). (**F**) Heart weight-to-body weight ratio at day 28 post-MI (n=4). Data are presented as the mean ±SEM.

Figure S6

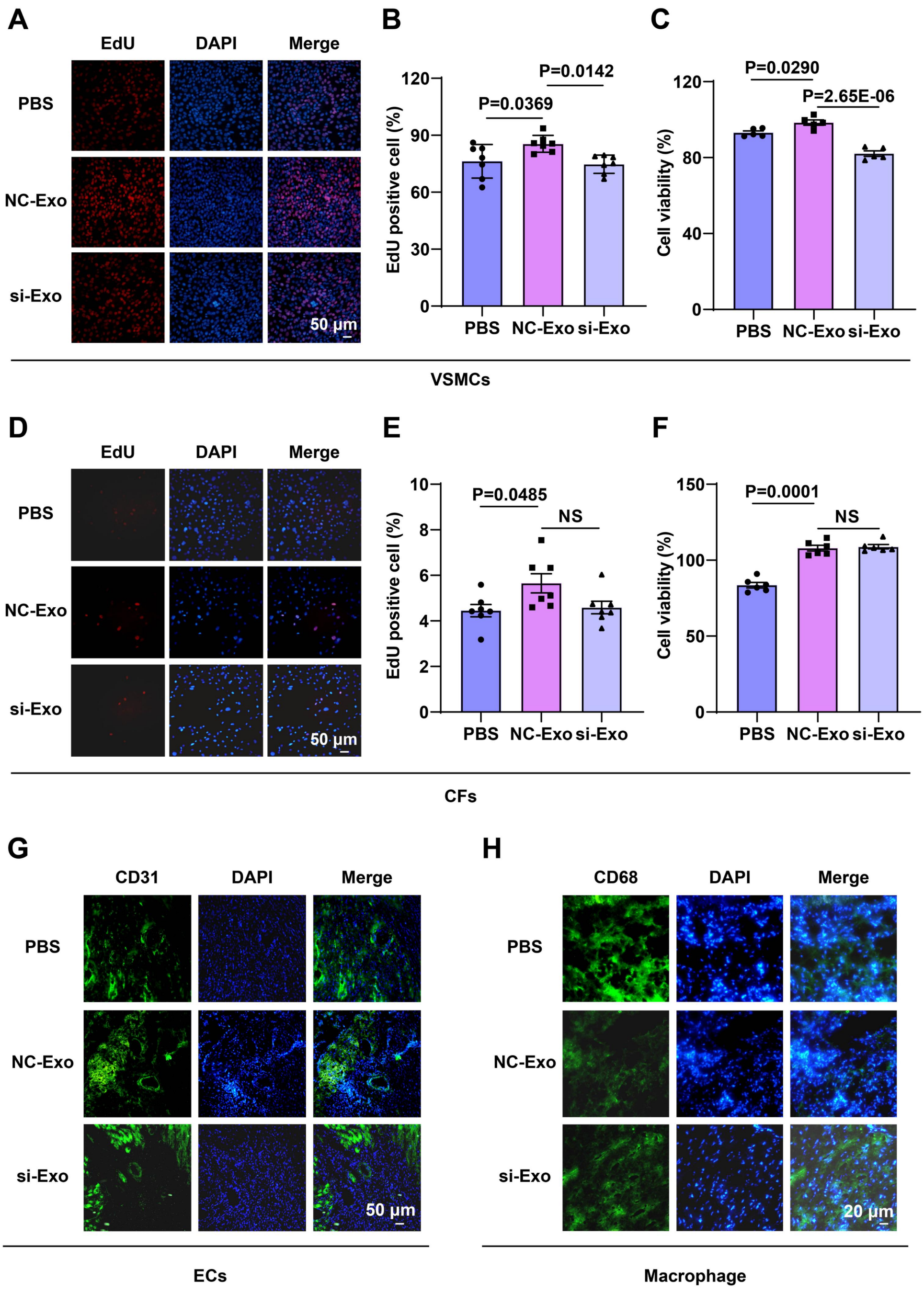

Figure S6. Exos regulate biological functions of various cardiac cell types. (**A, B**) The proliferation of VSMCs was detected by EdU incorporation. The cells were pre-treated with PBS, NC-Exo and si-Exo. Blue: nuclear staining (DAPI); Red: EdU staining (scale bar: 50 μm) (n=7). (**C**) The cell viability of VSMCs was detected by CCK8. The cells were pre-treated with PBS, NC-Exo and si-Exo (n=5). (**D, E**) The

proliferation of CFs was detected by EdU incorporation. The cells were pre-treated with PBS, NC-Exo and si-Exo. Blue: nuclear staining (DAPI); Red: EdU staining (scale bar: 50 μm) (n=7). (**F**) The cell viability of CFs was detected by CCK8. The cells were pre-treated with PBS, NC-Exo and si-Exo (n=6). (**G**) The angiogenesis at day 7 after MI was detected by CD31 immunofluorescence staining (n=3). (**H**) The macrophage infiltration in different treatment groups at day 7 after MI was detected by CD68 immunofluorescence staining (n=3). Data are presented as the mean ± SEM.

Figure S7

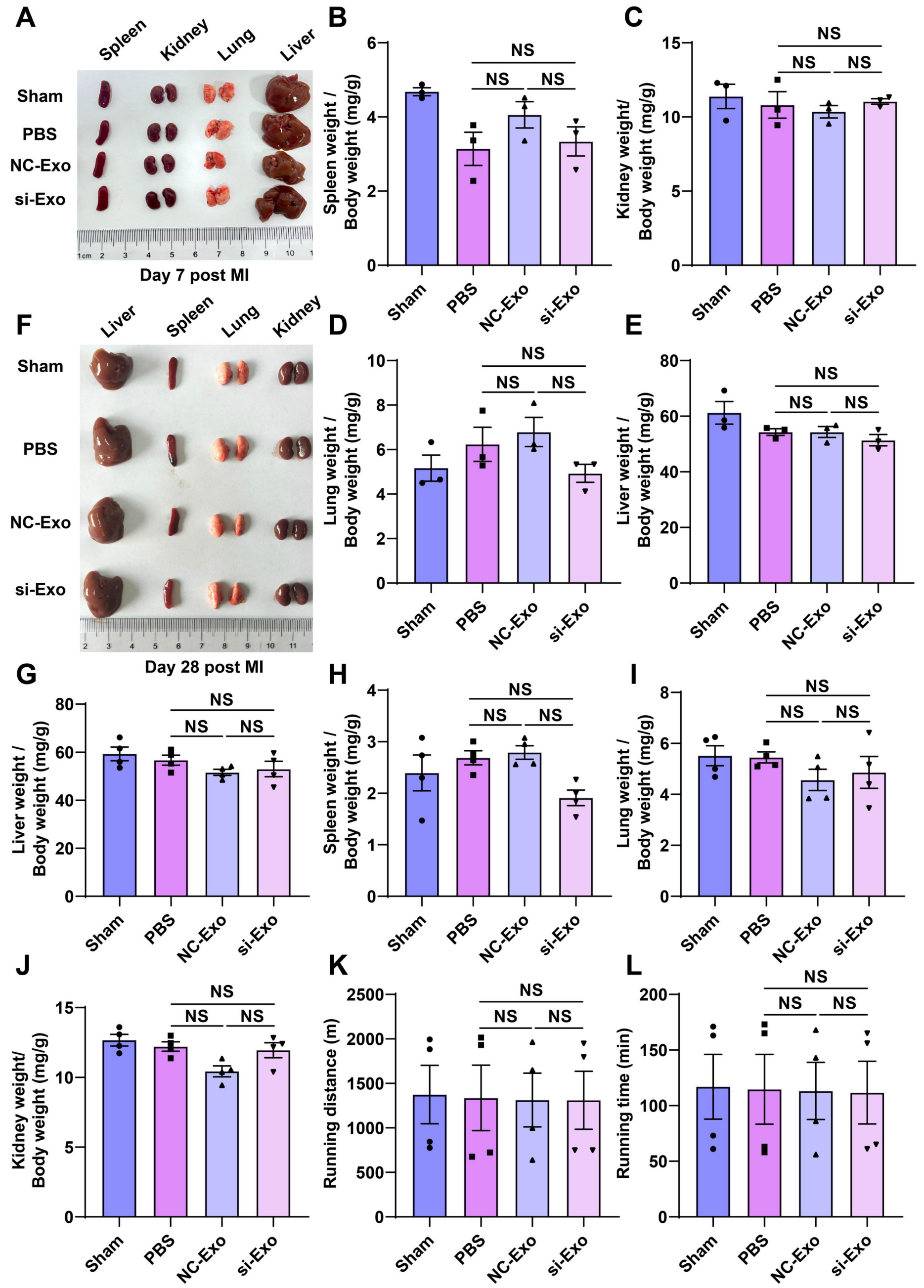

Figure S7. Exos do not have a significant impact on other organs. (**A**) Image of organ shape across the treatment group at day 7 post-MI (n=3). (**B-E**) Organ weight-to-body weight ratio at day 7 post-MI (n=3). (**F**) Image of organ shape across the treatment groups at day 28 post-MI (n=4). (**G-J**) Organ weight-to-body weight ratio at day 28 post-MI (n=4). (**K, L**) Running distance and running time at day 28 post-MI (n=4). Data are presented as the mean ± SEM.

Figure S8

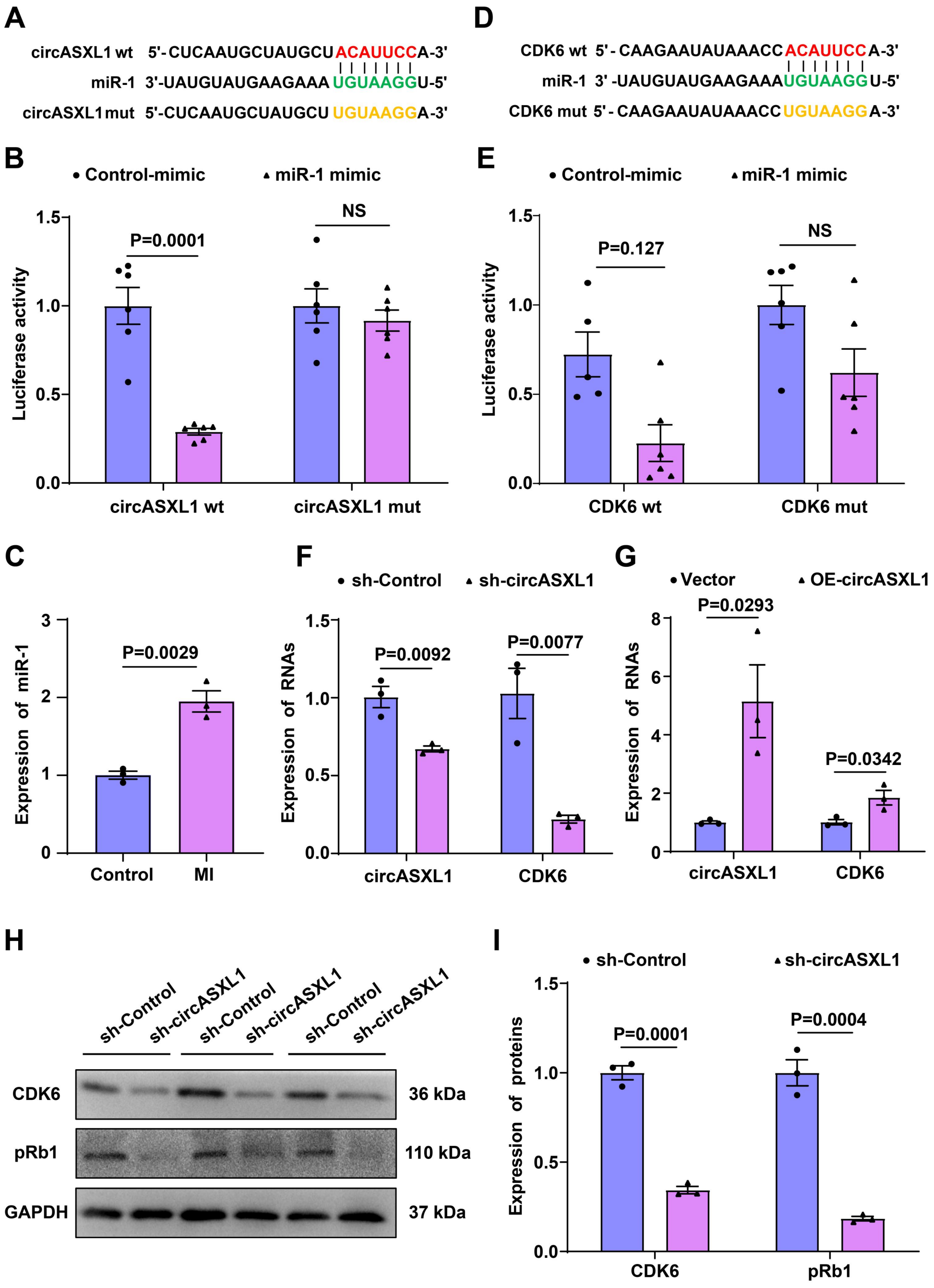

Figure S8. circASXL1 promotes cardiac repair by regulating the miR-1/CDK6 signaling pathway. (**A**) Illustration of putative complementary sites within circASXL1 and miR-1 by CircBank analysis. (**B**) Dual-luciferase reporter assay confirming the interaction between circASXL1 and miR-1 (n=6). (**C**) RT-qPCR analysis of miR-1 after MI (3 days after MI, n=3). (**D**) Bioinformatics analysis

identified CDK6 as a target for miR-1. (**E**) Dual-luciferase reporter assay confirming the interaction between miR-1 and CDK6 (n=6). (**F**) RT-qPCR analysis of the expression of circASXL1 and CDK6 in H9C2 transfected with sh-Control or sh-circASXL1 (n=3). (**G**) RT-qPCR analysis of the expression of circASXL1 and CDK6 in H9C2 transfected with vector plasmid or circASXL1-overexpression plasmid (n=3). (**H, I**) Western blot analysis of the expression of CDK6 or pRb1 in CMs transfected with sh-Control or sh-circASXL1 (n=3). Data are presented as the mean ± SEM.

Figure S9

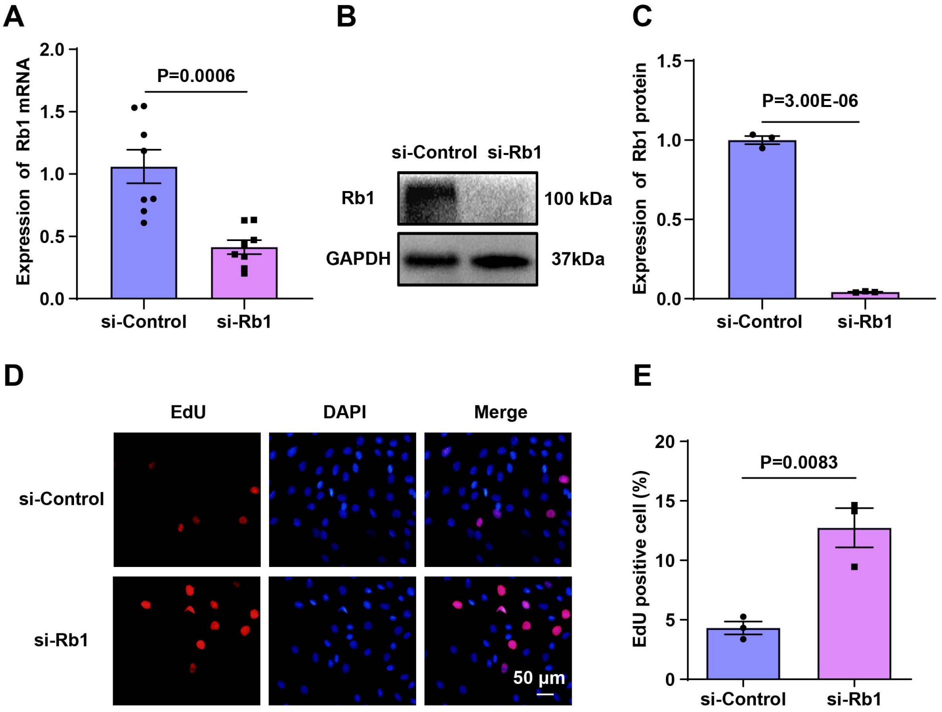

Figure S9. Inhibition of Rb1 expression promotes H9C2 (rat CMs) proliferation. (**A**) RT-qPCR analysis of Rb1 expression in H9C2 transfected with siRNA targeting Rb1 (si-Rb1) or control (si-Control) (n=8). (**B, C**) Western blot analysis of Rb1 expression in H9C2 transfected with siRNA targeting Rb1 (si-Rb1) or control (si-Control) (n=3). (**D, E**) The proliferation of CMs was detected by EdU incorporation. The cells were pre-treated with si-Control or si-Rb1. Blue: nuclear staining (DAPI); Red: EdU staining (scale bar: 50 μm) (n=3). Data are presented as the mean ± SEM.

Figure S10

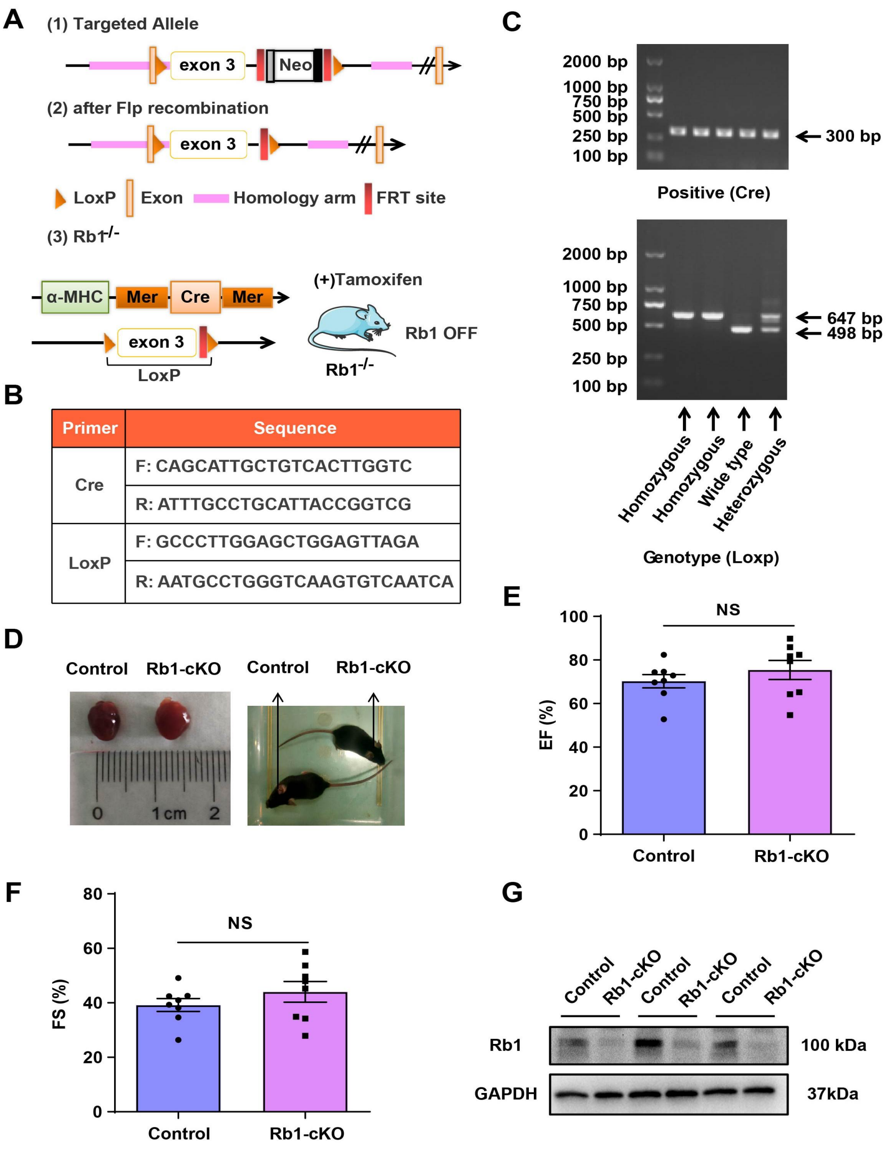

Figure S10. Construction of Rb1 conditional knockout flox mice and identification of genotype. (**A**) Construction strategy of cardiac-specific Rb1-cKO mice. (**B, C**) The results of genotype identification (Cre positive: 300 bp; Loxp-Homozygous: 647 bp; Loxp-Heterozygous: 498 bp and 647 bp; Loxp-Wide Type: 498 bp). (**D**) Image of Control and Rb1-cKO mice (left: cardiac shape; right: appearance). (**E, F**) Cardiac function was assessed by echocardiography in control and Rb1-cKO mice (n=8). (**G**) Western blot analysis of the expression of Rb1 in control and Rb1-cKO mice (n=3). Data are presented as the mean ± SEM.

Figure S11

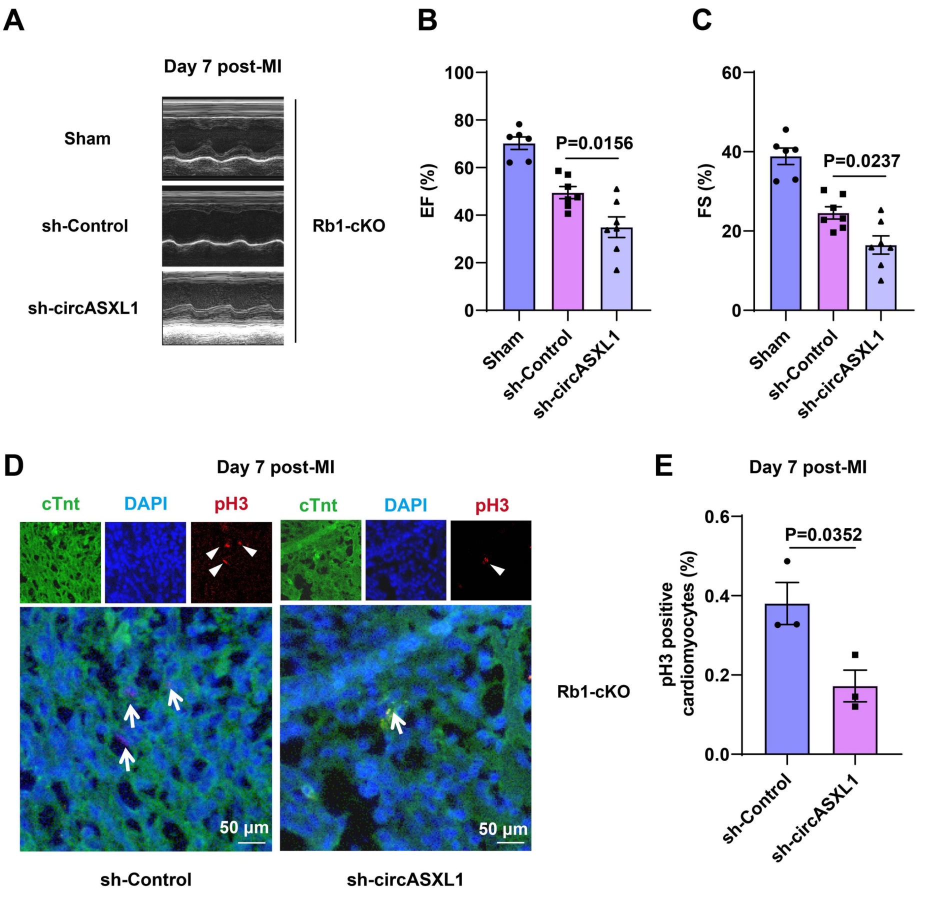

Figure S11. circASXL1 promotes proliferation of CMs in Rb1-cKO mice *in vivo*. (**A-C**) Cardiac function was assessed by echocardiography at 7 days post-injury (n = 6). (**D, E**) The proliferation of CMs was detected by pH3 immunofluorescence on frozen sections of the myocardium (n=3).

Figure S12

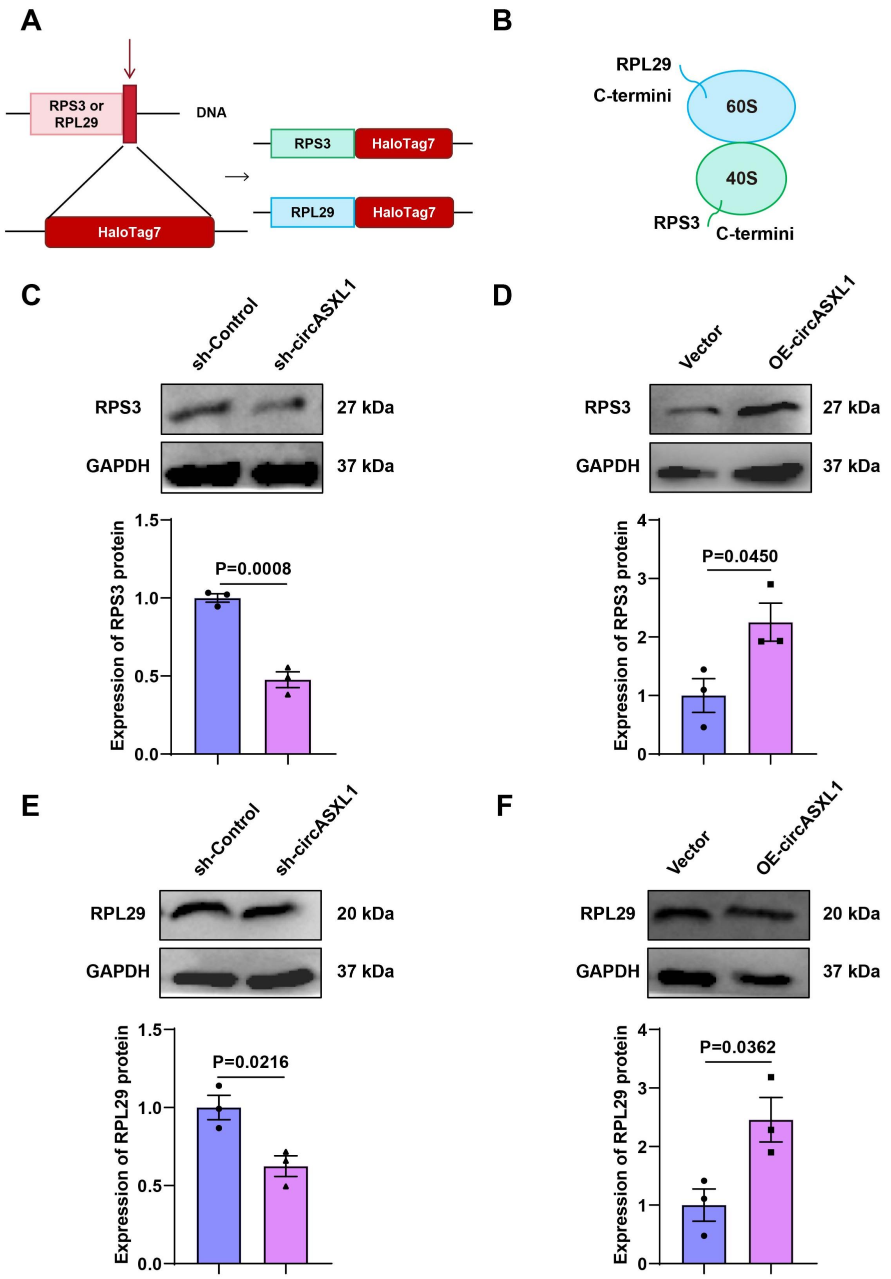

Figure S12. circASXL1 promotes Ribo-bio in CMs. (**A**) Generation of Ribo-Halo reporters to fuse Halo with the C-termini of RPS3 and RPL29. (**B**) RPS3 and RPL29 contain solvent exposed C-termini and are located far from the peptide exit tunnel based on the structure of an 80S complex. (**C-F**) The R-protein synthesis was analyzed by Western blot. The cells were pre-treated with sh-Control, sh-circASXL1, or vector plasmid and circASXL1-overexpression plasmid (n=3).

Figure S13

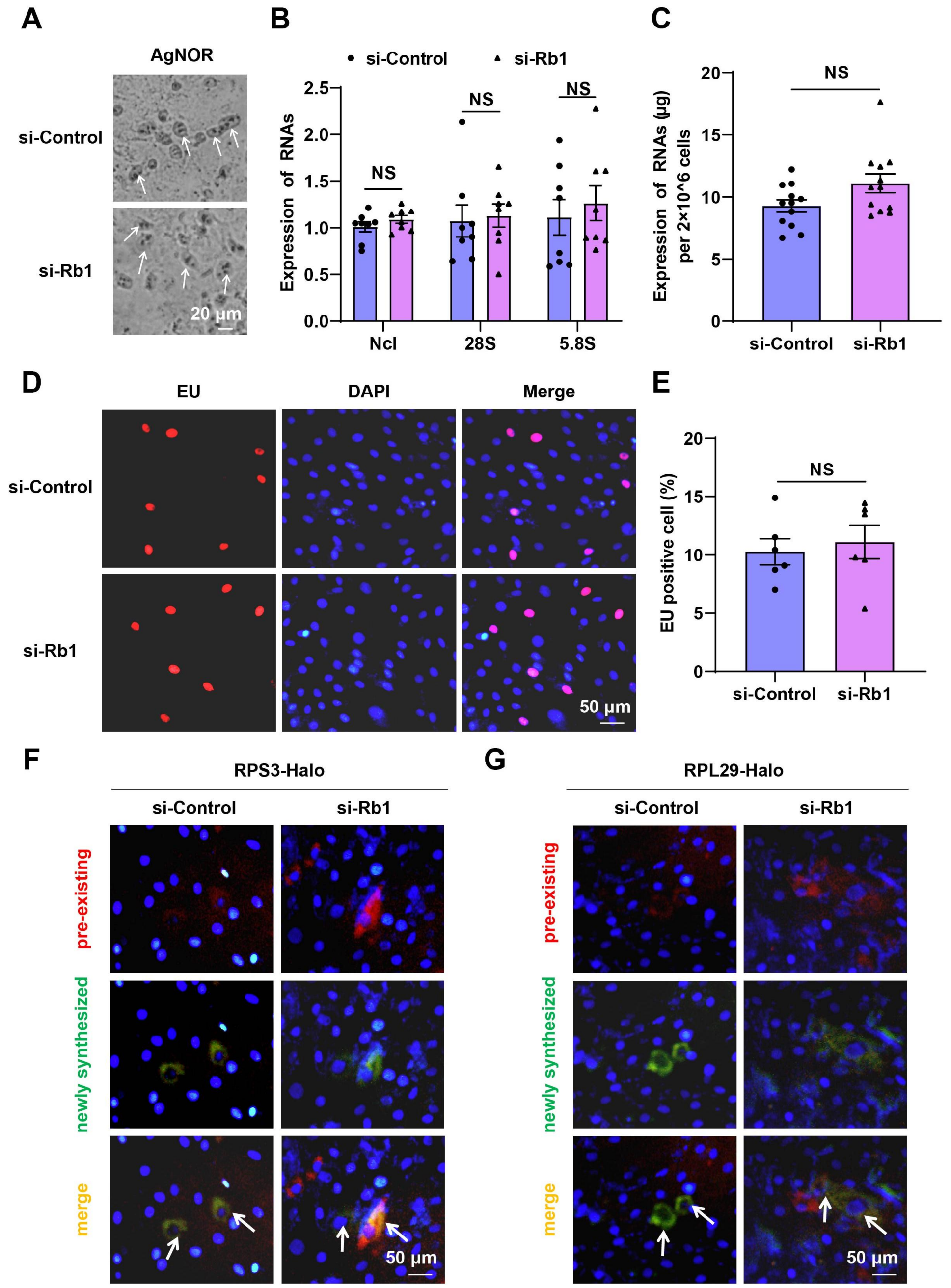

Figure S13. Rb1 does not regulate ribo-bio of H9C2 cell (rat CMs). (**A**) The Ribo-bio of H9C2 was detected by AgNOR staining (n=3). (**B**) RT-qPCR analysis showed that Rb1 siRNA treatment does not affect levels of Ncl, 28S, 5.8S transcript (n = 8). (**C**) The total RNA content of H9C2 was measured as an index of ribosomal content (n=12). (**D, E**) The rRNA synthesis of H9C2 was detected by EU incorporation. The cells were pre-treated with si-Control or si-Rb1. Blue: nuclear

staining (DAPI); Red: EU staining (scale bar: 50 μm) (n=6). (**F**) The Ribo-bio was detected by RRS3-Halo. The H9C2 were pre-treated with si-Control and si-Rb1. Blue: nuclear staining (DAPI); (scale bar: 50 μm) (n=3). (**G**) The Ribo-bio was detected by RRL29-Halo. The H9C2 were pre-treated with si-Control and si-Rb1. Blue: nuclear staining (DAPI); (scale bar: 50 μm) (n=3). Data are presented as the mean ± SEM.

Figure S14

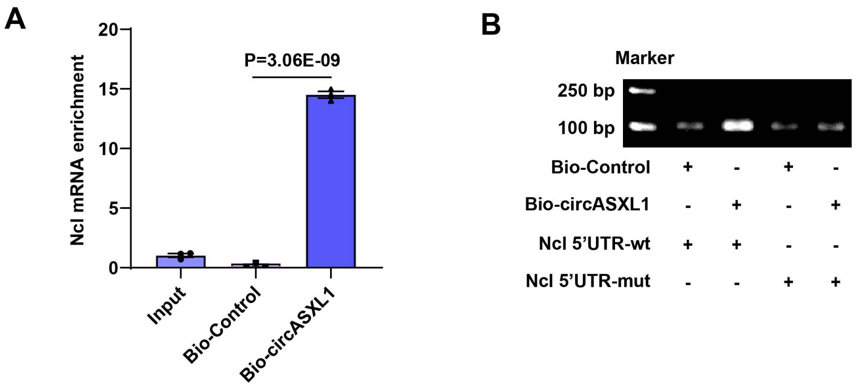

Figure S14. CircASXL1 binds to Ncl mRNA. (**A**) The interaction between circASXL1 and Ncl mRNA was confirmed by circRNA pulldown assay (n=3). (**B**) circRNA pulldown revealed that circASXL1 could bind to Ncl 5'UTR (n=3). Data are presented as the mean ± SEM.

Figure S15

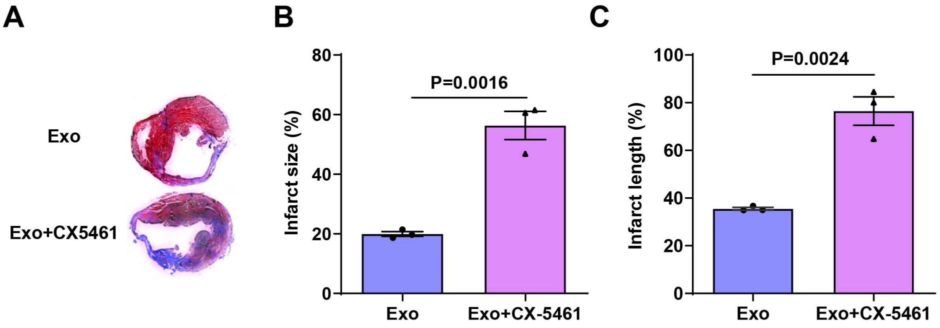

Figure S15. Exosome-derived circASXL1 inhibits myocardial fibrosis by enhancing Ribo-bio. (**A-C**) Representative Masson's trichrome-stained transverse sections and quantification of fibrotic scars in Exo and Exo+CX-5461 treated groups (n=3). Data are presented as the mean ± SEM.

Figure S16

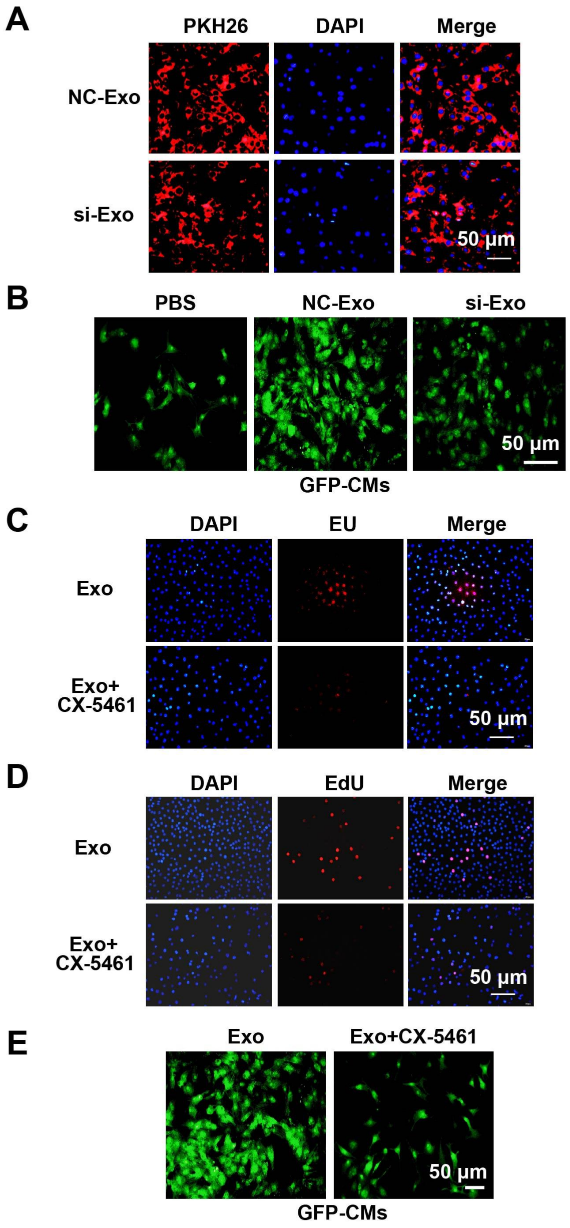

Figure S16. CX-5461 attenuates the beneficial effects of exosomes. (**A**) H9C2 **(**Rat CMs) were incubated with PKH26-labeled Exo for 24 h, and Exo uptake was detected by fluorescence microscopy. Blue: nuclear staining (DAPI); Red: PKH-26-Exo staining (scale bar: 50 μm) (n=5). (**B**) The primary CMs from GFP mice were treated with PBS, NC-Exo or si-Exo (n=3). (**C**) The Ribo-bio of H9C2 was detected by EU incorporation. The cells were pre-treated with exosome or CX-5461 and exosome. Blue: nuclear staining (DAPI); Red: EU staining (scale bar: 50 μm) (n=3). (**D**) The

proliferation of H9C2 was detected by EdU incorporation. The cells were pre-treated with exosome or CX-5461 and exosome. Blue: nuclear staining (DAPI); Red: EdU staining (scale bar: 50 μm) (n=5). (**E**) The primary CMs from GFP mice were treated with exosome or CX-5461 and exosome (n=3).

Table S1: Cardiac function measured by echocardiography 4 weeks post MI (Exosomes):

	Sham N=6	PBS N=6	Exo N=6	si-Exo N=6
**IVS;d (mm)**	0.73±0.02	0.48±0.04	1.10±0.11	0.60±0.05
**IVS;s (mm)**	1.00±0.06	0.47±0.06	1.29±0.06	0.69±0.06
**LVID;d (mm)**	2.85±0.18	4.44±0.15	4.10±0.25	4.70±0.23
**LVID;s (mm)**	1.85±0.22	3.88±0.11	3.32±0.27	4.13±0.27
**LVPW;d (mm)**	0.84±0.03	1.28±0.06	1.24±0.15	0.93±0.06
**LVPW;s (mm)**	1.01±0.04	1.41±0.07	1.42±0.11	1.04±0.10
**EF (%)**	65.42±6.05	26.94±1.07	40.18±3.93	28.67±2.93
**FS (%)**	35.56±4.39	12.44±0.56	19.46±2.13	13.38±1.47
**LV mass**	66.64±4.54	159.07±7.85	223.37±48.58	145.16±8.82
**LV mass corrected**	53.31±3.63	127.26±6.28	178.69±38.86	116.13±7.05
**LV Vol;d (µL)**	31.80±5.40	90.30±7.23	76.45±12.36	97.28±11.67
**LV Vol;s (µL)**	11.92±4.18	65.65±4.55	47.21±10.43	70.81±11.24

Data are mean ± SEM. IVS;d: interventricular septum thickness in diastole; IVS;s: interventricular septum thickness in systole; LVID;d: left ventricular end-diastolic diameter; LVID;s: left ventricular end-systolic diameter; LVPW;d: left ventricle posterior wall thickness in diastole/systole; LVPW;s: left ventricle posterior wall thickness in diastole/systole; EF: ejection fraction; FS: fractional shortening; LV Vol;d: left ventricular enddiastolic volume; LV Vol;s: left ventricular end-systolic volume.

Table S2: Cardiac function measured by echocardiography 1 weeks post MI (Rb1-cKO):

	Sham<br>N=6	Control<br>N=6	Rb1-cKO<br>N=6
**IVS;d (mm)**	0.75±0.06	0.89±0.06	0.93±0.09
**IVS;s (mm)**	0.10±0.08	1.04±0.09	1.22±0.12
**LVID;d (mm)**	2.56±0.18	3.89±0.27	3.52±0.24
**LVID;s (mm)**	1.68±0.19	3.35±0.27	2.85±0.25
**LVPW;d (mm)**	0.901±0.06	1.15±0.05	1.13±0.10
**LVPW;s (mm)**	1.04±0.09	1.19±0.09	1.18±0.10
**EF (%)**	64.78±5.16	26.52±4.81	40.89±3.36
**FS (%)**	34.78±4.31	12.26±2.45	19.53±1.77
**LV mass**	67.50±13.01	174.65±28.53	143.05±25.14
**LV mass corrected**	54.00±10.40	139.72±22.83	114.44±20.11
**LV Vol;d (µL)**	25.11±4.59	71.95±16.00	53.36±8.74
**LV Vol;s (µL)**	9.39±2.45	54.17±13.20	32.77±6.90

Data are mean ± SEM. IVS;d: interventricular septum thickness in diastole; IVS;s: interventricular septum thickness in systole; LVID;d: left ventricular end-diastolic diameter; LVID;s: left ventricular end-systolic diameter; LVPW;d: left ventricle posterior wall thickness in diastole/systole; LVPW;s: left ventricle posterior wall thickness in diastole/systole; EF: ejection fraction; FS: fractional shortening; LV Vol;d: left ventricular enddiastolic volume; LV Vol;s: left ventricular end-systolic volume.

Table S3: Cardiac function measured by echocardiography 4 weeks post MI (Rb1-cKO):

	Sham<br>N=6	Control<br>N=6	Rb1-cKO<br>N=6
**IVS;d (mm)**	0.66±0.04	0.67±0.02	0.60±0.05
**IVS;s (mm)**	1.15±0.06	0.84±0.05	0.81±0.07
**LVID;d (mm)**	3.04±0.28	4.62±0.18	4.48±0.27
**LVID;s (mm)**	1.97±0.23	3.97±0.17	3.80±0.26
**LVPW;d (mm)**	0.95±0.05	1.35±0.12	1.18±0.04
**LVPW;s (mm)**	1.02±0.09	1.39±0.13	1.35±0.09
**EF (%)**	65.49±5.19	30.36±2.53	32.23±2.88
**FS (%)**	35.59±3.99	14.27±1.28	15.21±1.48
**LV mass**	79.07±11.70	203.20±11.35	164.66±14.67
**LV mass corrected**	63.26±9.36	162.56±9.08	131.73±11.74
**LV Vol;d (μL)**	38.68±7.98	99.61±9.51	93.74±11.97
**LV Vol;s (μL)**	13.89±3.61	69.58±7.35	64.40±10.06

Data are mean ± SEM. IVS;d: interventricular septum thickness in diastole; IVS;s: interventricular septum thickness in systole; LVID;d: left ventricular end-diastolic diameter; LVID;s: left ventricular end-systolic diameter; LVPW;d: left ventricle posterior wall thickness in diastole/systole; LVPW;s: left ventricle posterior wall thickness in diastole/systole; EF: ejection fraction; FS: fractional shortening; LV Vol;d: left ventricular enddiastolic volume; LV Vol;s: left ventricular end-systolic volume.

Table S4: Cardiac function measured by echocardiography 4 weeks post MI (CX-5461):

	Sham<br>N=6	NC-Exo<br>N=6	Exo+CX-5461<br>N=6
**IVS;d (mm)**	0.75±0.03	0.83±0.06	0.59±0.02
**IVS;s (mm)**	0.98±0.06	0.88±0.06	0.58±0.04
**LVID;d (mm)**	2.64±0.26	3.91±0.13	4.17±0.19
**LVID;s (mm)**	1.80±0.27	3.42±0.12	3.78±0.17
**LVPW;d (mm)**	0.96±0.10	1.47±0.20	1.19±0.08
**LVPW;s (mm)**	1.15±0.04	1.64±0.25	1.17±0.06
**EF (%)**	64.09±5.43	27.49±1.33	20.56±1.66
**FS (%)**	34.03±3.65	12.57±0.67	9.25±0.81
**LV mass**	65.96±8.46	198.10±35.44	146.21±9.57
**LV mass corrected**	52.77±6.77	158.46±28.35	116.97±7.66
**LV Vol;d (μL)**	27.60±7.33	66.89±5.52	78.32±8.42
**LV Vol;s (μL)**	11.24±4.22	48.51±4.20	62.22±6.85

Data are mean ± SEM. IVS;d: interventricular septum thickness in diastole; IVS;s: interventricular septum thickness in systole; LVID;d: left ventricular end-diastolic diameter; LVID;s: left ventricular end-systolic diameter; LVPW;d: left ventricle posterior wall thickness in diastole/systole; LVPW;s: left ventricle posterior wall thickness in diastole/systole; EF: ejection fraction; FS: fractional shortening; LV Vol;d: left ventricular enddiastolic volume; LV Vol;s: left ventricular end-systolic volume.

Table S5. List of Ligands used for Ribo-Halo.

Ligands	Catalog No.	Company	Dilution
HaloTag® R110Direct™ Ligand	G3221	Promega	50 nM
HaloTag TMR (5mM) Ligand	G8251	Promega	100 nM

Table S6. Oligonucleotides used for qPCR.

mRNA	Forward primer (5'-3')	Reverse primer (5'-3')
**Species (Human)**		
GAPDH	GGTGGTCTCCTCTGACTTCAACA	GTTGCTGTAGCCAAATTCGTTGT
circASXL1	GGACTTCCCCTCTCGCATG	TCCTTCTGCCTCTATGACCTG
circSMARCA5	CTCCAAGATGGGCGAAAG	TGTGTTGCTCCATGTCTAATCA
circCCDC66	TTGAGGAACGAGACAGACGAC	TGCAGTTCTTGTTTCACAGCAC
circHECTD1	GCTTCAATTGTCCTGTAATGGCA	CCGGCGTCCTCCTTTAGTTT
circMYLK	GCCTTGTGATTCATGCTGTCC	CACATCCCCCATGGTCTTCT
**Species (Mouse)**		
GAPDH	AAATGGTGAAGGTCGGTGTG	TGAAGGGGTCGTTGATGG
circASXL1	GAGGAGGAGAAGGGCTGTTTT	CTGTTTTGGTGTCATTGGAGCA
circSMARCA5	GGACACAGAGTCCAGTGTTTA	TCCCAATTTTGTTCAGGTTCTGAT
circCCDC66	GCTGTATCACACGGTCCGAA	CTCCTGTTTAATGGCGCTGC

circHECTD1	AACTTAGGCGTATTTGGGAGC	ACATAGTCGTCATCCCAGGC
circMYLK	GGGCAAATACACCTGTGAAGC	TGTGTGACGAGGCAAACAGT
CDKN2a	CGCAGGTTCTTGGTCACTGT	TGTTCACGAAAGCCAGAGCG
Rb1	AAAGCTGCGCTTTGACATCG	ATGAGCCAGGAGTCTGGTGT
CDK6	CTGTGGAAGAAAAGTGCAGAGA	TAGACGGACCGACCTTCTCG
Ncl	CAGGGAACAGTTTGGTGGGT	GCTGAGTGCCTTCAGCTACA
RNA Pol I	CAAAGAGGCTCCACTCAGGG	GGGAGTGTTCTGGTCTGGTG
47S Pre-rRNA	GCTTGTTTCTCCCGATTGC	CGCGAACAACTGAGAAAGT
28S	AGCCGACTTAGAACTGGTGC	GGCAGAAATCACATCGCGTC
5.8S	CTTAGCGGTGGATCACTCGG	GCAAGTGCGTTCGAAGTGTC
**Species (Rat)**		
GAPDH	CAACGGGAAACCCATCACCAT	AGATGATGACCCTTTTGGCCCC
Rb1	TCCCAGCGGAGTCCAAATTC	TCCCGAGGGTCTACAGTGTT
Ncl	TTCATTACCCGCCGATCCAG	TGGACTCTCCGTGGGTTTTG
RNA Pol I	CCCTGCTTTGAGCCTTACGA	TGGCCTGATACCGGTAAGGA
47S Pre-rRNA	GTTCCGCTCACACCTCAGAT	CAAGTGCGTTCGAAGTGTCG
28S	AGCCGACTTAGAACTGGTGC	GGCAGAAATCACATCGCGTC
5.8S	CTTAGCGGTGGATCACTCGG	GCAAGTGCGTTCGAAGTGTC

Table S7. List of antibodies

Epitope	Catalog No.	Company	Use	Dilution	Source
**Primary antibodies**					
GAPDH	sc-32233	Santa Cruz Biotechnology	WB	1:1000	Mouse
CD63	sc-5275	Santa Cruz Biotechnology	WB, FC	1:1000, 1:500	Mouse
TSG101	sc-7964	Santa Cruz Biotechnology	WB	1:1000	Mouse
cTnT	ab8295	Abcam	IF	1:200	Mouse
Aurora B	ab2254	Abcam	IF	1:200	Rabbit
pH3	ab47297	Abcam	IF	1:200	Rabbit
CDKN2a	32050	Signalway Antibody	WB	1:1000	Rabbit
CDK6	3136S	Cell Signaling Technology	WB	1:1000	Mouse
Rb1	ab181616	Abcam	WB	1:1000	Rabbit
Ncl	14574S	Cell Signaling Technology	WB	1:1000	Rabbit
RNA Pol I	sc-48385	Santa Cruz Biotechnology	WB	1:1000	Rabbit
RPS3	9538S	Cell Signaling Technology	WB	1:1000	Rabbit
RPL29	15799-1-AP	Proteintech Group	WB	1:1000	Rabbit
**Secondary antibodies**					
Anti-mouse HRP	A0216	Beyotime Biotechnology	WB	1:2000	Goat
Anti-rabbit HRP	A0208	Beyotime Biotechnology	WB	1:2000	Goat
Goat Anti-Mouse IgG H&L (Alexa Fluor® 488)	ab150113	Abcam	IF	1:200	Goat
Donkey Anti-Rabbit IgG H&L (Alexa Fluor® 647)	ab150075	Abcam	IF	1:200	Donkey
